# Transcriptomic Analysis of Tea Plant Responding to Drought Stress and Recovery

**DOI:** 10.1371/journal.pone.0147306

**Published:** 2016-01-20

**Authors:** Sheng-Chuan Liu, Ji-Qiang Jin, Jian-Qiang Ma, Ming-Zhe Yao, Chun-Lei Ma, Chun-Fang Li, Zhao-Tang Ding, Liang Chen

**Affiliations:** 1 Tea Research Institute of the Chinese Academy of Agricultural Sciences, Key Laboratory of Tea Plant Biology and Resources Utilization, Ministry of Agriculture, Hangzhou, Zhejiang, China; 2 Guizhou Tea Research Institute, Guiyang, Guizhou, China; 3 Tea Research Institute, Qingdao Agricultural University, Qingdao, Shandong, China; NBPGR, INDIA

## Abstract

Tea plant (*Camellia sinensis*) is an economically important beverage crop. Drought stress (DS) seriously limits the growth and development of tea plant, thus affecting crop yield and quality. To elucidate the molecular mechanisms of tea plant responding to DS, we performed transcriptomic analysis of tea plant during the three stages [control (CK) and during DS, and recovery (RC) after DS] using RNA sequencing (RNA-Seq). Totally 378.08 million high-quality trimmed reads were obtained and assembled into 59,674 unigenes, which were extensively annotated. There were 5,955 differentially expressed genes (DEGs) among the three stages. Among them, 3,948 and 1,673 DEGs were up-regulated under DS and RC, respectively. RNA-Seq data were further confirmed by qRT-PCR analysis. Genes involved in abscisic acid (ABA), ethylene, and jasmonic acid biosynthesis and signaling were generally up-regulated under DS and down-regulated during RC. Tea plant potentially used an exchange pathway for biosynthesis of indole-3-acetic acid (IAA) and salicylic acid under DS. IAA signaling was possibly decreased under DS but increased after RC. Genes encoding enzymes involved in cytokinin synthesis were up-regulated under DS, but down-regulated during RC. It seemed probable that cytokinin signaling was slightly enhanced under DS. In total, 762 and 950 protein kinases belonging to 26 families were differentially expressed during DS and RC, respectively. Overall, 547 and 604 transcription factor (TF) genes belonging to 58 families were induced in the DS vs. CK and RC vs. DS libraries, respectively. Most members of the 12 TF families were up-regulated under DS. Under DS, genes related to starch synthesis were down-regulated, while those related to starch decomposition were up-regulated. Mannitol, trehalose and sucrose synthesis-related genes were up-regulated under DS. Proline was probably mainly biosynthesized from glutamate under DS and RC. The mechanism by which ABA regulated stomatal movement under DS and RC was partly clarified. These results document the global and novel responses of tea plant during DS and RC. These data will serve as a valuable resource for drought-tolerance research and will be useful for breeding drought-resistant tea cultivars.

## Introduction

Drought is a major factor influencing the growth and development of crops; thus, it affects crop quality and yield worldwide. Given the changes in climate, especially global warming, and the increasing demand for water for non-agricultural use, breeding elite cultivars with high drought resistance and recoverability is an important target for crop breeders. Some recent studies have documented and explained plant system responses to drought stress (DS) [1,2]. The morphological and physiological mechanisms of drought tolerance in many plants have been fully elucidated, but the global transcriptome profiles of some plants in response to DS and rehydration are still lacking.

The molecular responses of plants to DS include perception, signal transduction, gene expression, and ultimately metabolic changes that lead to stress tolerance [3]. Various regulatory and functional genes are involved in these processes, since drought tolerance is a complex multigenic trait [4]. For example, abscisic acid (ABA) has been shown to play crucial roles in regulating the drought response, and its metabolic pathway involves multiple steps and genes [5]. 9-*cis*-Epoxycarotenoid dioxygenase (NCED) is a key enzyme for ABA biosynthesis, but only one of the five NCED genes in *Arabidopsis*, *AtNCED3*, was significantly triggered by DS [6]. The pyrabactin resistance (PYR)/PYR-like(PYL)/regulatory components of ABA receptor (RCAR) -type ABA receptors, type 2C protein phosphatases (PP2C), and sucrose non-fermenting 1-related protein kinase 2 (SnRK2) constitute the core regulatory network of ABA signaling, which can activate a series of transcription factors (TFs) to cope with DS [5]. Also involved in the responses to drought and rewatering is a myriad of genes involved in the metabolism and signaling of other phytohormones, osmolyte metabolism, regulation of antioxidant activity and stomatal movement, etc [7]. However, the functions of most these genes and their regulatory networks have remained elusive. Therefore, further research is required to explore the gene networks involved in drought response and tolerance, and to identify new drought-related genes.

Tea plant (*Camellia sinensis*) is one of the most popular beverage crops in the world [8]. Drought is a major constraint for the growth, yield and quality of tea plant. It was reported that drought reduced tea production by 14–33%, and caused 6–19% plant mortality [9]. As previously reported, tea plants adapt to resist DS through a series of physiological responses such as osmotic adjustment, scavenging reactive oxygen species (ROS), and phytohormone regulation [8,10,11]. A set of drought-responsive genes in tea plant were identified using cDNA-amplified fragment length polymorphism or suppression subtractive hybridization analyses [12–14]. However, compared with other woody species, less is known about the drought tolerance mechanisms of tea plant at the genome-wide transcriptional level.

Recently, rapid advances in RNA sequencing (RNA-Seq) and associated bioinformatics tools have provided revolutionary tools for transcriptomic research on plants [15]. For example, the global transcriptomic profiles of drought responses have been surveyed in *Populus* [16], *Phaseolus vulgaris* [17], and *Ammopiptanthus mongolicus* [18] using this approach. Therefore, the aim of this study was to identify drought-responsive genes, and to deeply elucidate the signaling, regulatory and metabolic mechanisms that operate during drought and rewatering. To achieve these aims, five *C*. *sinensis* libraries were subjected to RNA-Seq analyses. The experimental materials were 10-year-old plants of the drought-tolerant tea cultivar ‘Ningzhou 2’, an elite clone selected from Jiangxi Province, China [8]. The plants were subjected to DS for eight days and then allowed to recover (RC) after rewatering. The data obtained in this study not only contribute to our understanding of the molecular mechanisms of this species in response to DS and rewatering, but will also be useful for breeding drought-resistant tea plant.

## Materials and Methods

### Stress treatment and RNA isolation

The experiment was conducted at the China National Germplasm Hangzhou Tea Repository (latitude, 30° 10′ 808ʺ N; longitude, 120° 05′ 370ʺ E; altitude, 27 m a.s.l.) from July to August 2013. The tea plants were subjected to severe DS and then allowed to recover (RC) after rewatering in field conditions [8]. We selected 10-year-old plants of the drought-tolerant tea cultivar ‘Ningzhou 2’ for this work, because our previous study had documented the drought-resistance characteristics of this cultivar [8]. The control (CK) tea plants were sampled on July 22, a cloudy day when the soil had 79% of field moisture capacity. The plants under DS were sampled from July 26 to July 31. As in our previous study, drought-stressed plants were considered to be rehydrated at 96 h after rewatering [8]. Under CK, during DS, and under RC, ‘two and a bud’ samples (one young shoot with two leaves and a bud) were collected from 20 plants, immediately frozen in liquid N_2_, and stored at −80°C. Leaf materials were collected once every 4 days from 17:00 to 17:30.

Total RNA was extracted using the RNeasy Plant Mini Kit according to the manufacturer’s instructions (Qiagen, Hilden, Germany) and treated with RNase-free DNase II (Takara, Dalian, China). The quantity and purity of total RNA were assessed using a NanoDrop ND-2000 spectrophotometer (NanoDrop Technologies, Wilmington, DE, USA) and 1% formaldehyde-agarose gel electrophoresis. RNA samples with A260/A280 values ranging from 1.9 to 2.1 and A260/A230 ratios greater than 2.0 were chosen. The cDNA libraries were constructed from approximately 25 μg of total RNA (with an RNA concentration of ≥ 650 ng/μL) from the CK, DS, and RC samples. The remaining RNA was used for quantitative real-time polymerase chain reaction (qRT-PCR) analyses.

### Illumina cDNA library preparation and sequencing

After total RNA extraction and DNase I treatment, poly (A) mRNA was first enriched using magnetic beads with oligo (dT). After mixing with fragmentation buffer, the mRNA was cut into short fragments, and then cDNA was synthesized using these cleaved mRNA fragments as the templates. Short fragments were purified and resolved with EB buffer for end-repair and single nucleotide A (adenine) addition. Then, the short fragments were connected with adaptors. Suitable fragments were selected as templates for PCR amplification. During the quality control steps, Agilent 2100 Bioanaylzer (Agilent Technologies, Santa Clara, CA, USA) and the StepOnePlus^™^ Real-Time PCR System (Applied Biosystems, Foster City, CA, USA) were used to analyze the size and quality of the sample libraries. The libraries were sequenced with the Illumina HiSeq^™^ 2000 to generate raw data with an average read length of 100 bp.

### Preprocessing and *de novo* assembly

Raw reads produced from HiSeq^™^ 2000 sequencing were preprocessed to remove reads with adaptors, reads containing more than 5% unknown bases, and low-quality reads (>20% of the bases with a quality score of ≤ 10). The filtered reads were *de novo* assembled by Trinity software [19] to construct contigs with large volumes of RNA-Seq reads. The contigs were realigned to construct unigenes using Trinity software. The paired-end reads were used to fill intra-scaffold gaps to obtain sequences with the least number of nonsense sequences and that could not be extended at either end. After clustering and assembly using TGICL software [20], a non-redundant unigene set from all five assembled datasets was finally constructed. After assembly, a series of sequential BLASTx (E-value ≤ 10^−5^) searches against non-redundant protein (NR), Swiss-Prot, Kyoto Encyclopedia of Genes and Genomes (KEGG), and Clusters of Orthologous Groups (COG) were performed. The best alignments were used to decide the direction of unigene sequences. The sequence orientation of the unigenes that were not found in the above databases was determined using ESTScan software [21].

### Unigene annotation and classification

To obtain information on the expression and functional annotation of the unigenes, all assembled unique sequences were aligned to NR, Swiss-Prot, KEGG, COG, and The Arabidopsis Information Resource (TAIR) using BLASTx, and to the non-redundant nucleotide (Nt) sequence database with BLASTn (E-value ≤ 10^−5^). The protein with the highest sequence similarity to each given unigene was retrieved. Based on NR annotations, gene ontology (GO) annotations were assigned to unigenes using Blast2GO software [22], and then GO functional classification was performed using WEGO software [23] to understand the distribution of gene functions. The unigenes were also aligned to the COG database to predict and classify gene functions. The unigene products related to metabolism in the cellular processes group were analyzed and annotated according to the KEGG database.

### Protein coding region prediction and transcription factor analysis

To predict protein coding sequences (CDSs), unigenes were first aligned by BLASTx (E-value ≤ 10^−5^) to protein databases in the following order of priority: NR, Swiss-Prot, KEGG, and then COG. Unigenes that had been aligned to a higher-priority database were not aligned to a lower-priority database. The coding-region sequences (5′–3′) of unigenes were decided based on the highest ranks in the BLAST results. The coding-region sequences were translated into amino acid sequences (5′–3′) with the standard codon table. TFs were predicted according to protein sequences obtained from CDSs prediction. The TFs were identified and classified by searching PlantTFDB3.0 (the plant TF database 3.0) [24] with E-values ≤ 10^−5^.

### Expression and KEGG analysis for differentially expressed unigenes

The expression levels of unigenes were calculated as fragments per kilobase of exon per million fragments mapped (FPKM) [25]. Gene expression profiles from RNA-Seq data were analyzed using Expectation-Maximization (RSEM) software [26] bundled with the Trinity package. Differentially expressed unigenes [false discovery rate (FDR) <10^−3^, E-values ≤ 10^−5^, |log_2_^ratio^| ≥ 1] among the five libraries were identified. Heat maps were generated and hierarchical clustering was conducted using Cluster 3.0 software [27].

To further clarify the biological functions of differentially expressed genes (DEGs), GO term and KEGG pathway enrichment analyses of DEGs were conducted with BINGO (*P*-values ≤ 0.05 after Bonferroni correction) [28].

### Quantitative PCR analysis

Although RNA-Seq is a highly efficient sequencing procedure to screen for DEGs, errors still occur because the transcriptome is assembled from billions of short RNA-Seq reads [29]. To validate the reliability of the expression profiles observed in the RNA-Seq data, 20 genes were randomly selected for qRT-PCR analyses using a Power SYBR Premix Ex TaqTM II Kit (Perfect RealTime, Takara, Dalian, China) with an ABI 7500 Real-Time PCR system (Applied Biosystems, Foster City, CA, USA) according to the manufacturer’s instructions. The glyceraldehyde-3-phosphate dehydrogenase (GAPDH) gene (KA295375.1, *C*. *sinensis*) was utilized as an internal control. The relative expression value was calculated by the delta-delta CT method and expressed as the fold change relative to expression in the null controls (expression = 1)[30]. Primers used in the qRT-PCR analyses were listed in S1 Table. Three technical replicates per sample were analyzed to ensure statistical credibility.

## Results

### RNA-Seq and *de novo* assembly

As we reported previously, DS was imposed gradually by withholding water for up to eight days, until the soil volumetric moisture content (SWC) decreased to approximately 12.5%, compared to about 21.0% in CK [8]. Our result was largely consistent with that of Maritim *et al*. (2013), who reported that tea plant suffered from severe DS with SWC less than 53.0% of that in CK [11]. Then, SWC was returned to the normal moisture level by rewatering. Changes in leaf morphological and physiological traits were also monitored, ensuring that the stress levels were adequate and equivalent for this species [8]. RNA-Seq of five libraries (one library for CK, two repeats for DS, and two repeats for RC) resulted in 407.96 million reads with more than 98% exhibiting a quality score of Q20 (99% accuracy) (Table 1). These data were then deposited in the National Center for Biotechnology Information (NCBI) with accession number of PRJNA297732 (http://www.ncbi.nlm.nih.gov/bioproject/297732). In total, 378.08 million high-quality trimmed reads were *de novo* assembled into contigs by the software Trinity (Table 1 and S1A Fig). The contigs were assembled into 59,674 unigenes with an average length of 760 bp and an N50 length of 1,123 bp (Table 1). All unigenes were longer than 200 bp and 17.1% (10,340) of them were longer than 1,000 bp (S1B Fig).

**Table 1:** Overview of sequencing and assembly.

Sequences	CK	DS1	DS2	RC1	RC1	Total
Total raw reads (million)	90.77	68.50	93.63	75.83	79.23	407.96
Total clean reads (million)	84.25	63.48	86.72	70.28	73.35	378.08
Clean bases (Gb)	8.43	6.35	8.67	7.03	7.34	37.82
Q20 (%)	98.42	98.40	98.45	98.41	98.39	98.41
N (%)	0.00	0.00	0.00	0.00	0.00	0.00
GC (%)	46.65	46.29	46.00	46.01	47.10	46.41
N50 of contigs (bp)	291	309	228	238	213	398
Mean length of unignes (bp)	516	542	437	437	420	760
N50 of unigenes (bp)	781	870	593	599	566	1,213

CK: control samples; DS1, DS2: drought treated samples; RC1, RC2: rewatering treated samples.

### Functional annotation and classification of unigenes

**Fig 1:**
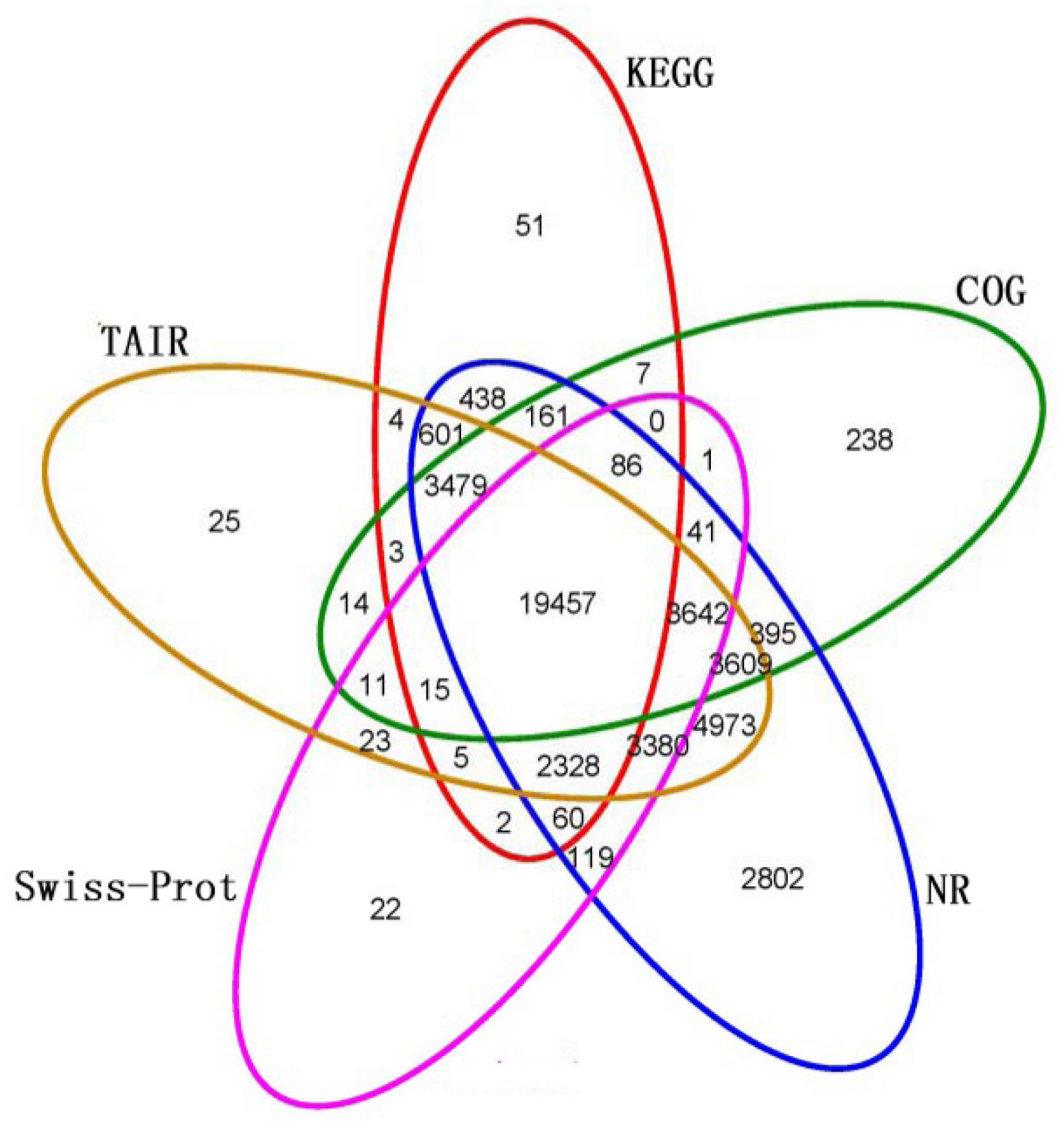
Venn diagram showing BLAST results of *Camellia sinensis* transcriptome against five databases. *De nov*o reconstructed transcript sequences were used in BLAST searches against public databases: KEGG, COG, NR, Swiss-Prot, and TAIR. Number of unigenes with significant hits (E-value ≤10^−5^) against five databases is shown at each intersection of Venn diagram.

A total of 26,696 (44.1%), 31,159 (51.4%), 45,570 (75.2%), 29,191 (48.2%) and 41,569 (68.6%) unigenes had significant hits (E-value ≤10^−5^) in KEGG, COG, NR, Swiss-Prot, and TAIR, respectively. Of the 59,674 high-quality unique sequences, 48,089 (80.59%) unigenes significantly matched a sequence in at least one of the five databases and 19,457 unigenes showed similarity to proteins in all of the five databases (Fig 1).

**Fig 2:**
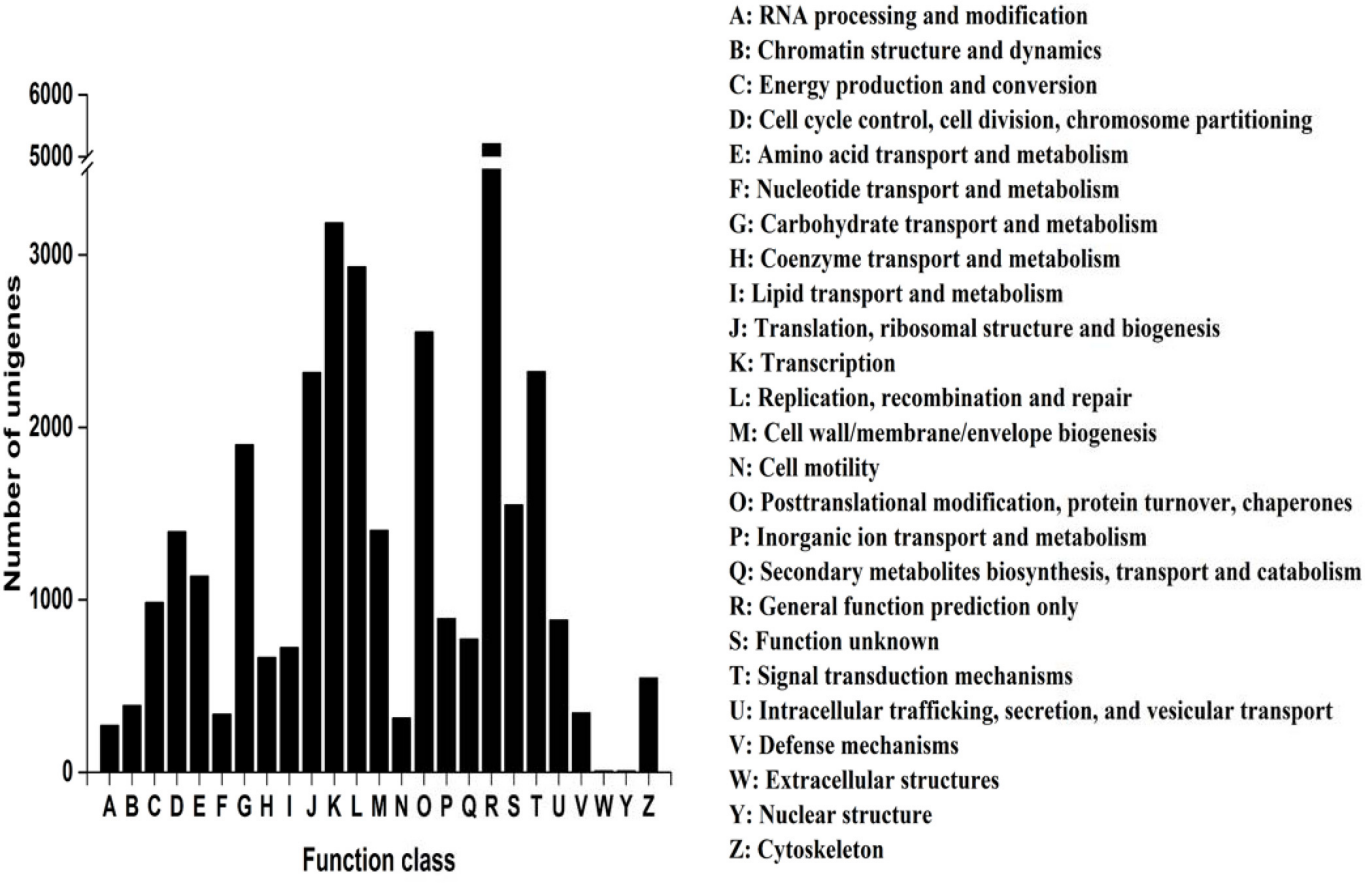
COG functional classification of *Camellia sinensis* transcriptome. In total, 31,159 unigenes with significant homologies in COG database (E-value ≤10^−5^) were classified into 25 COG categories. Capital letters on x-axis indicate COG categories as listed at right of histogram; y-axis indicates the number of unigenes.

Within the *C*. *sinensis* unigene set, 31,159 (51.4%) unigenes were categorized (E-value ≤10^−5^) in 25 COG clusters (Fig 2). The five largest categories were: 1) general function predictions only (16.7%), 2) transcription (10.2%), 3) replication; recombination and repair (9.4%), 4) post-translational modification, protein turnover, chaperones (8.2%), and 5) signal transduction mechanisms (7.5%).

**Fig 3:**
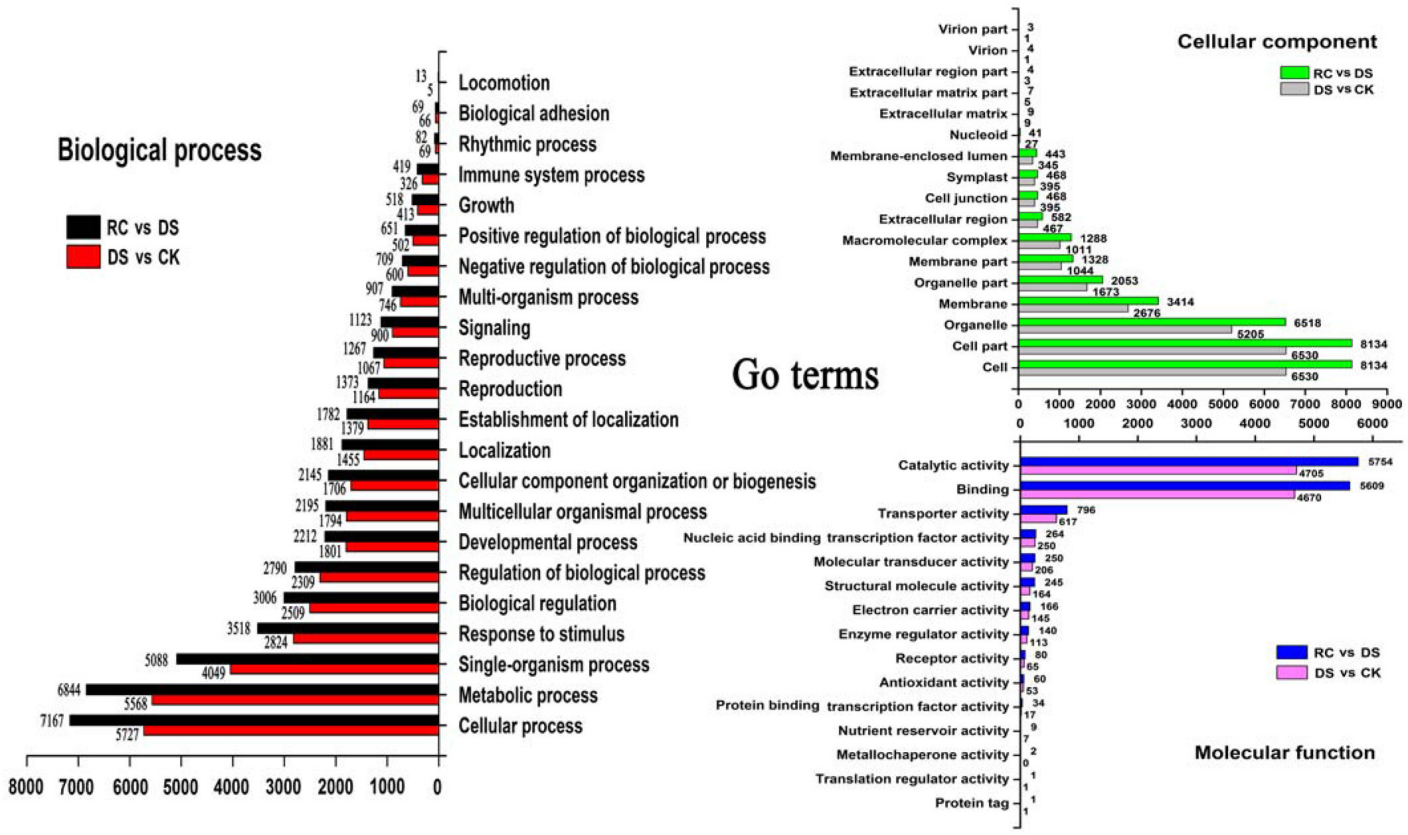
Function classifications of GO terms of *Camellia sinensis* transcripts. Based on highscore BLASTx matches in NR plant proteins database, a total of 35,304 unigenes were classified into three main GO categories and 31 sub-categories. Number of genes in a specific category within the main category is shown on y-axis; number of unigenes is shown in x-axis.

Based on classification of GO terms, a total of 35,304 unigenes were assigned with at least one GO term. The largest subgroups in the biological process category were cellular process, metabolic process, single-organism process, and response to stimulus (Fig 3). In the cellular component category, the largest subgroups were cell, cell part, organelle, and membrane. In the molecular function category, catalytic activity and binding accounted for 85.12% and 84.73% of all unigenes in the DS vs. CK and RC vs. DS libraries, respectively.

### Protein coding sequence prediction

A total of 45,775 unigenes CDSs were identified by the BLASTx protein database searches described above. Of the unigenes with CDSs, the majority (24,655 unigenes; 53.9%) were longer than 500 bp and 10,249 unigenes were longer than 1,000 bp (S2 Fig). Using the ESTScan program, we assigned another 1,709 unigene CDSs that could not be aligned to above databases, and obtained the length frequency distributions of these unigene CDSs and their corresponding amino acid sequences (S2 Fig).

### Metabolic pathway analysis of differentially expressed genes under drought stress and recovery

**Fig 4:**
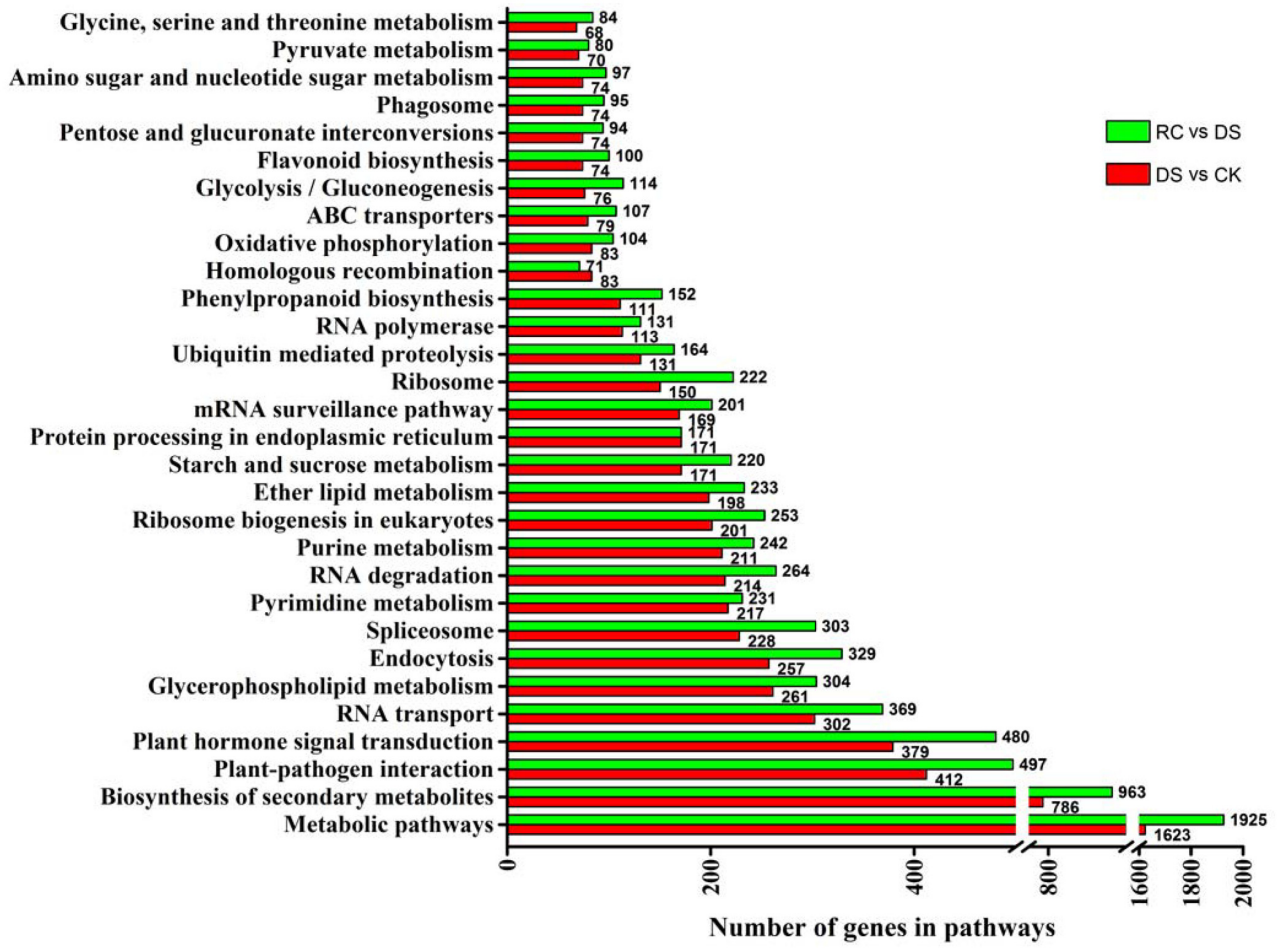
Largest number of unigenes for 30 main KEGG pathways in tea plant during and after drought stress. Number of genes in each pathway is shown on x-axis; pathway categories are shown on y-axis.

Totally 6,959 (26.1%) and 8,525 (31.9%) of the 26,696 unigenes had significant matches in KEGG. These genes were assigned to 127 KEGG pathways in the DS vs. CK and RC vs. DS libraries. Among the KEGG pathways, the largest groups were metabolic pathway, biosynthesis of secondary metabolites, plant—pathogen interaction, and plant hormone signal transduction (Fig 4). The KEGG pathway enrichment analysis showed that 13 and 7 of the KEGG pathways were significantly enriched under DS and RC, respectively (S3 Fig). Under DS, the metabolic pathway group had the largest number of unique sequences (1,623 unigenes) (ko01100). These sequences encoded products that were involved in various metabolic pathways; for example, energy metabolism, and metabolism of carbohydrates, glycans, nucleotides, amino acids, and lipids [31]. By contrast, pathways associated with biosynthesis, especially biosynthesis of secondary metabolites, were mainly enriched during RC. Our data revealed that these metabolic and biosynthetic pathways may be crucial for the response to dehydration and rehydration in tea plant.

Based on GO functional annotations and pathway enrichment analysis of the DEGs, the results showed that the DEGs involved in major biological processes and metabolic pathways mainly encoded regulatory proteins (TFs, protein kinases, protein phosphatases, signaling molecules, and other regulatory proteins) and functional proteins (osmotin, membrane proteins, metabolic enzymes, channel proteins, chaperones, transport proteins, and various proteases) (Figs 3 and 4).

### Differentially expressed genes among the three stages

**Fig 5:**
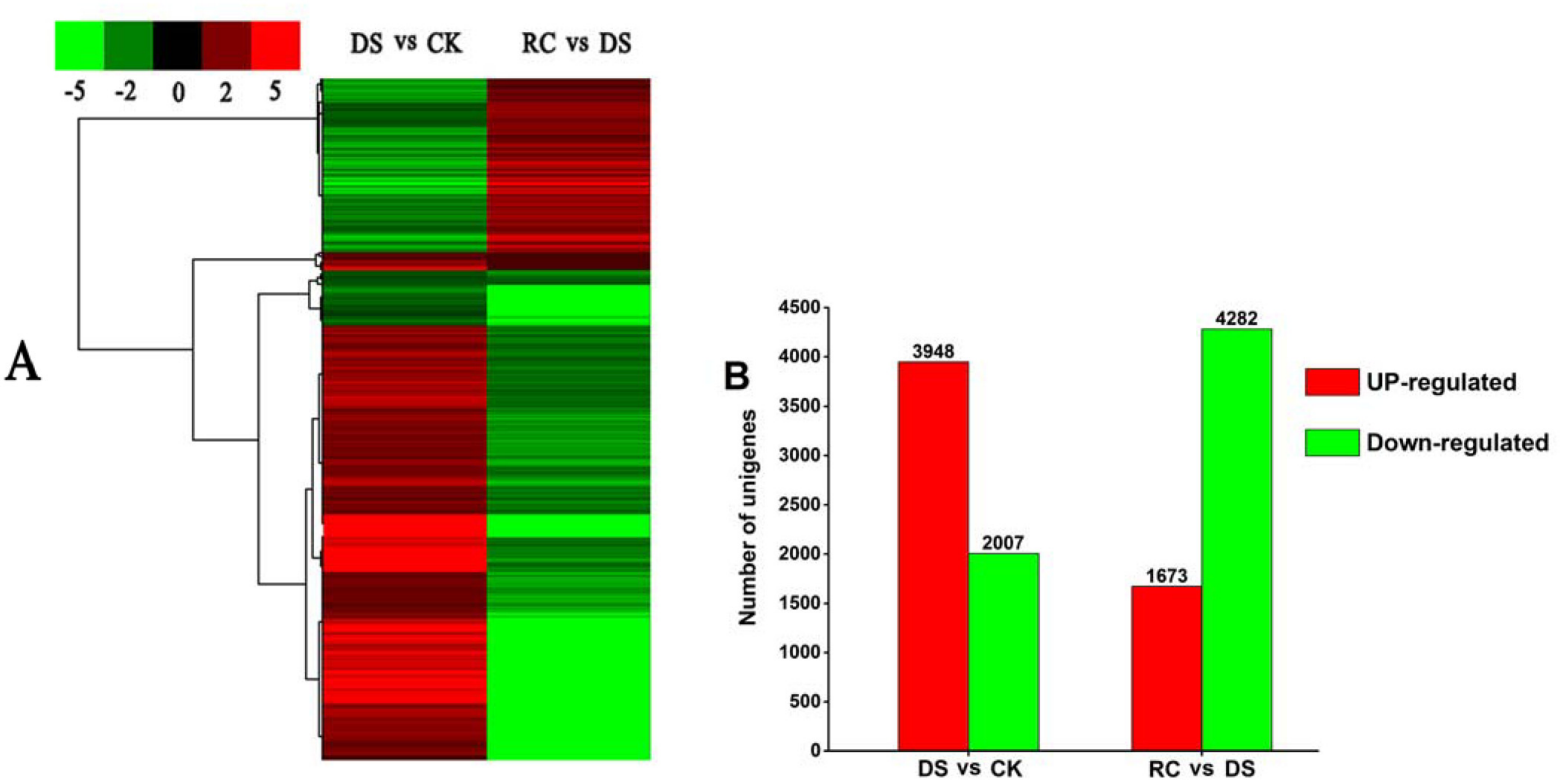
Changes in transcript levels of differentially expressed genes (DEGs) in tea plant during and after drought stress. (A) Heat-maps of all DEGs. Columns and rows in heat maps represent samples and DEGs, respectively. (B) Number of up- and down-regulated DEGs under dehydration and rehydration (FDR<10^−3^, *P*-value<10^−5^, |log_2_ratio|≥1).

The expression levels of 5,955 DEGs differed significantly among the three stages (Fig 5A); 1,517 DEGs that were down-regulated under DS were then up-regulated after RC, while the opposite trend was observed for 3,792 DEGs, but 490 DEGs and 156 DEGs were down-regulated and up-regulated during the three stages respectively (Fig 5A). In the DS vs. CK library, there were a total of 3,948 up-regulated DEGs and 2,007 down-regulated DEGs; in the RC vs. DS library, there were 1,673 up-regulated DEGs and 4,282 down-regulated DEGs in total (Fig 5B).

### Unigene validation and expression analysis

**Fig 6:**
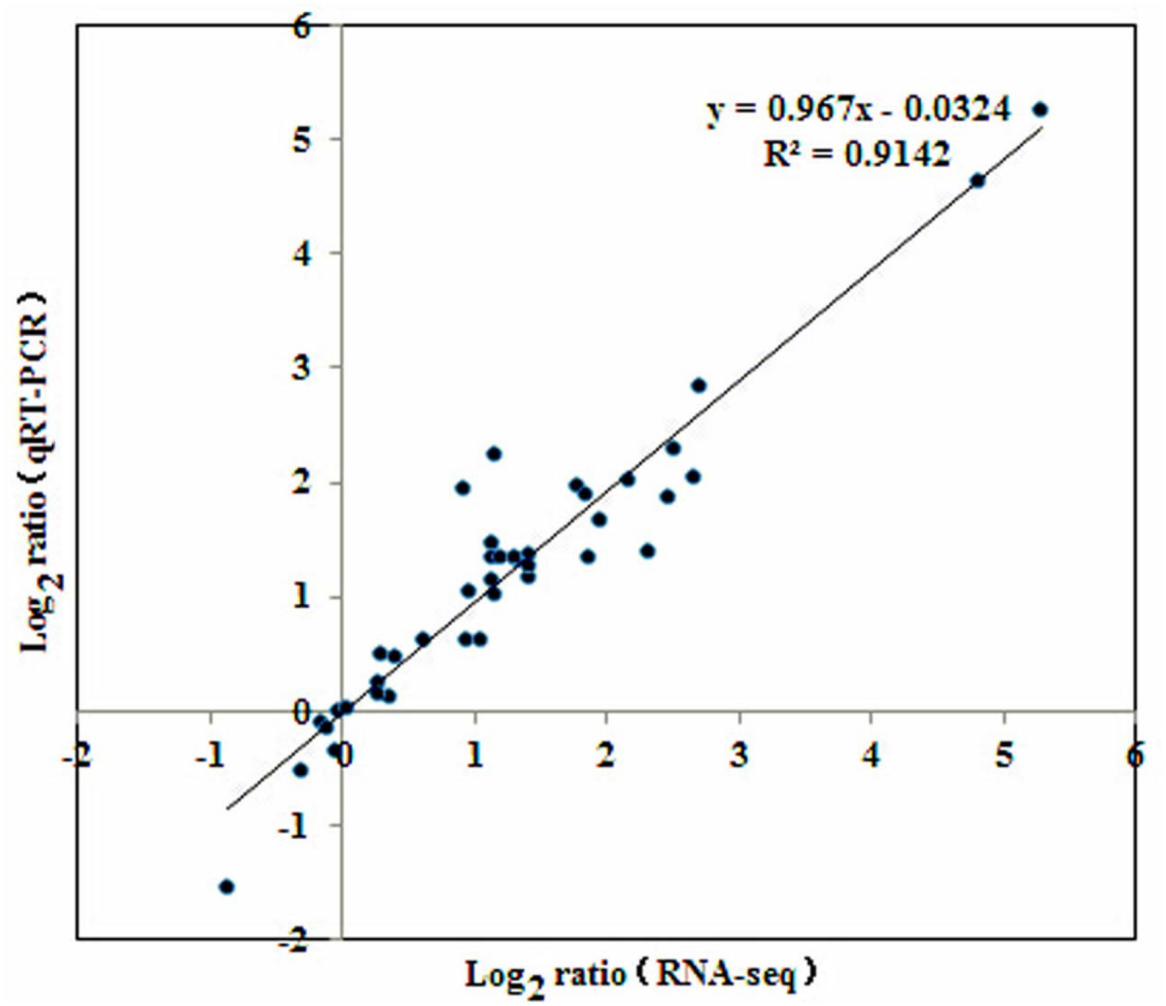
Consistency between RNA-Seq data and qRT-PCR data. Relationship between RNA-Seq data (x-axis) and qRT-PCR data (y-axis) using log_2_^ratio^ measure of transcript levels of 20 genes.

To confirm RNA-Seq results, qRT-PCR was conducted on 20 randomly selected DEGs based on transcriptional profile analysis. These DEGs involved in signaling, metabolism, transcriptional regulation, and physiological responses (S4 Fig). These unigenes determined by qRT-PCR shared similar expression tendency with those from RNA-Seq data (R^2^ = 0.91; Fig 6). Detailed comparisons between qRT-PCR and RNA-Seq results were shown in S4 Fig.

### Unigenes related to phytohormone metabolism and signaling during and after drought stress

Under DS, two key genes (*NCED1* and *NCED4*) of ABA biosynthesis were up-regulated; 12 genes (*PYL4*, *PYL8*, *PP2C1-6*, *SnRK2*.*2*, *SnRK2*.*3*, *SnRK2*.*5*, *SnRK2*.*6*) coding the core component of ABA signal transduction were also induced (S5A Fig). In addition, ABA key degradation enzyme gene *CYP707A* (cytokinin trans-hydroxylase), ABA sugar ester (ABA-GE) degrading enzyme gene *BG1* (β-D-glucopyranosyl abscisate β-glucosidase) and nicotinamide adenine dinucleotide phosphate oxidase gene *RbohD* (respiratory burst oxidase homolog D) were triggered. After rehydration, ABA synthesis and signal transduction related genes, as well as CYP707A gene were inhibited. Isochorismate synthase (ICS) pathway for salicylic acid (SA) synthesis was probably activated under DS, while phenylalanine ammonia-lyase (PAL) pathway was likely to be employed under RC (S5B Fig). It seemed probable that the dependent-nonexpressor of pathogenesis-related gene 1(*NPR1*) SA signaling pathway was inhibited, whereas the independent-*NPR1* pathway was activated by a regulator, suppressor of npr1-1, constitutive 1 (SNC1). In general, the expression levels of genes encoding jasmonic acid (JA) precursors [phospholipase A1 (PLA1), lipoxygenase 2 (LOX2), LOX3, allene oxide synthase (AOS), allene oxide cyclase (AOC)] decreased under DS, while the expression levels of genes encoding JA intermediates [12-oxophytodienoic acid reductase (OPR), OPC-8:0 CoA ligase 1 (OPCL1), acyl-CoA oxidase 2 (ACOX2)] increased, inducing enhancement of signal transduction. However, the opposite trend was observed during RC (S5C Fig). The expression of ethylene (ET) synthesis-related genes [*ACS* (1-aminocyclopropane-1-carboxylate synthase) and *ACO* (aminocyclopropanecarboxylate oxidase)] and signal transduction-related genes [*ETR* (ethylene receptor), *EIN2* (ethylene-insensitive protein 2), and *EIN3*] were also induced by drought (S5D Fig). Tea plant probably employed the 3-indoleacetaldoxime (IAOx) and indole-3-pyruvic acid (IPA) pathway for indole-3-acetic acid (IAA) synthesis under DS, while after rehydration the 3-indoleacetonitrile (IAN) pathway was mainly used (S5E Fig). Under DS, the expression levels of cytokinins (CKs) synthesis-related genes were slightly increased and decreased following rehydration (S5F Fig). Genes involved in IAA signaling were generally down-regulated under DS but up-regulated after RC (S5E Fig). Unexpectedly, genes involved in cytokinin signaling were slightly up-regulated under DS, and did not change significantly after RC (S5F Fig).

### Protein kinases, protein phosphatases, and transcription factors responding to dehydration and rehydration

Totally 762 and 950 protein kinases belonging to 26 families were found to be differentially regulated under DS and RC respectively (S6 Fig). In total, 53 and 81 differentially expressed protein phosphatases were observed under DS and RC respectively. These protein phosphatases were clustered into two families: the protein tyrosine phosphatase mainly including mitogen-activated protein kinase phosphatases (MKPs), and serine/threonine-protein phosphatase including PP2C, PP2A and PP1 (S6 Fig). Most members of 12 protein kinase families were activated by drought (S6 Fig). At the same time, most of the protein phosphatase genes were also up-regulated. After rehydration, the expression levels of protein phosphatase genes decreased. Correspondingly, the expression levels of protein kinase genes reduced. However, most members of eight protein kinases were up-regulated after rewatering.

In total, 547 and 604 TF genes were identified as DEGs in the DS vs. CK and RC vs. DS libraries, respectively (|log_2_^ratio^| ≥1). These TFs were classified into 58 families based on their putative DNA-binding and kinase domains (S7 Fig). Under DS, 260 TF genes were up-regulated and 287 were down-regulated. After RC, 276 TF genes were up-regulated and 328 were down-regulated. Most members of the 12 TF gene families were up-regulated under DS. These up-regulated genes included those encoding basic leucine zipper (bZIP), squamosa promoter binding protein-like (SPL), *Arabidopsis* response regulator (ARR), heat stress TF (HSF), WRKY, homeobox-leucine zipper protein (HD-ZIP), NAC domain-containing protein (NAC), scarecrow-like protein (SCL), myeloblastosis oncoprotein (MYB), APETALA2/ethylene-responsive element-binding protein (AP2/EREBP), basic helix-loop-helix protein (bHLH) and zinc-finger protein TFs. After rehydration, most members of the AP2/EREBP, bHLH, HD-ZIP, MYB, SCL, and SPL gene families were still up-regulated. In addition, most members of three TFs families [domain-containing transcription repressor (B3), growth-regulating factor (GRF), and TCP] were up-regulated.

### Changes in non-structural carbohydrate and proline metabolism in response to drought stress and recovery

The ribulose-1,5-bisphosphate carboxylase oxygenase (Rubisco) gene was down-regulated under DS and then up-regulated after rewatering (S8A Fig). Under DS, two key starch synthesis genes [*AGPase* (ADP-glucose pyrophosphorylase) and *SS* (starch synthase)] were inhibited, whereas three starch-degrading genes [*AAMY* (α-amylase), *BAMY* (β-amylase), and *SP* (starch phosphorylase)] were induced. In contrast, sucrose, threhalose, and mannitol synthesis-related genes [*UDPGase* (UDP-glucose pyrophosphorylase), *SPS* (sucrose-phosphate synthase), *TPS* (trehalose phosphate synthase), *TPP* (trehalose phosphatases), *M6PR* (mannose-6-phosphate reductase), and *M1PP* (mannose-1 phosphate phosphatase)] were up-regulated, while catabolism-related genes for these osmolytes were down-regulated, except for trehalase (TRE) gene, which was slightly up-regulated. Under RC, starch synthesis-related genes were up-regulated and starch decomposition-related genes were down-regulated. The opposite trend was observed for genes related to mannitol, trehalose, and sucrose biosynthesis and metabolism; that is, during RC, the genes related to their biosynthesis were down-regulated, while those related to their metabolism were up-regulated. As to proline metabolism under DS, the transcript levels of pyrroline-5-carboxylate synthetase (P5CS) gene, pyrroline-5-carboxylate reductase (P5CR) gene, and γ-glutamyl phosphate reductase (GRR) gene increased (S8B Fig). However, there were no significant changes in the expression level of ornithine amino transferase (OAT) gene during and after DS.

## Discussion

Original signal (drought and rewatering stimuli) by plant sensing cells perception, signaling and transport induced a series of drought-related gene expression and protein synthesis, and then plant optimally changed physiological and biochemical metabolism of recipient tissue to cope with DS and fast recover normal growth after rehydration [5,7]. In comparison to Gupta *et al*. (2012, 2013) and Das *et al*. (2012) studies [12–14], more DEGs were found and annotated in our study, which allowed us to further elucidate drought-resistance mechanisms of tea plant.

There were several ways to ensure the accuracy of the RNA-Seq data. Our previous morphological and physiological analyses could more accurately indicate the degree of stress and the reliability of the samples [8]. In the present study, the raw reads were processed by removing reads containing adapter, ploy-N and low quality reads. Q20 (>98%), GC-content and sequence duplication level of the clean data were calculated. After excluding the data generated from poor libraries and filtering low-quality reads, the 378.08 million (about 9-fold coverage) high-quality reads were used for *de novo* assembly. The assembled unigenes were extensively annotated, and these were further filtered for identifying differentially expressed genes. Additionally, the qRT-PCR results showed that all of randomly selected DEGs exhibited similar expression kinetics to those obtained from the RNA-Seq analysis.

### Drought and rehydration signal transduction of in tea plant

Phytohormones have crucial roles under drought and rewatering [32]. The DS response in plants involves four main phytohormones; ABA, SA, JA, and ET [7]. Two other phytohormones, IAA and CKs, regulate many aspects of plant growth and development and play essential roles in RC after DS. The results of our study provided details of the potential metabolism and signaling of these phytohormones during and after DS.

We found that most of the genes related to ABA biosynthesis and signaling were up-regulated under DS, and the transcript level of *CYP707A* also increased (S5A Fig). This is consistent with our earlier studies [8], which reported that ABA concentrations rapidly and significantly increased in tea plant leaves under DS. ABA is stored in its glucose ester form (ABA-GE). The BG1gene was up-regulated under DS, indicating that ABA-GE was converted into ABA. In our study, four SnRK2 genes were up-regulated under DS, indicating DS enhanced ABA responses. In addition, several lines of evidence have suggested that RbohD can be phosphorylated by SnRK2; thereby, it activates Ca^2+^ signaling and regulates stomatal movement in response to DS [33,34].

The ICS and PAL pathways are two distinct enzymatic pathways for SA biosynthesis in higher plants [35]. A significant increase in SA levels in tea plant leaves under DS has also been reported in our previous studies [8]. In the present study, it seemed probable that the ICS and PAL pathway were mainly used to synthesize SA in tea plant under DS and during RC respectively (S5B Fig). *NPR1* acts downstream of SA as a crucial regulator of the SA signaling pathway [36]. Overexpression of *NPR1* has been shown to enhance resistance to multiple diseases in diverse plant species, for example, cotton (*Gossypium hirsutum*) [37] and apple (*Malus × domestica*) [38]. Under DS, *NPR1*, TGA (TGA) gene (a TF gene downstream of *NPR1*) and genes encoding pathogenesis-related (PR) proteins (TGA-target genes) were down-regulated, probably leading to reduced disease resistance in tea plant (S5B Fig). In contrast, gene *SNC1* was up-regulated under DS. *SNC1* has been identified as a constitutively expressed PR gene [39]. These results suggested that the *NPR1*-independent resistance pathway was activated by SNC1 in tea plant under DS.

Our results suggested that many intermediate products in the peroxisome were generated from phosphatidylcholine metabolism, which provided the main blocks for JA biosynthesis in tea plant under DS. Also, the up-regulation of a drought-induced JA-amino synthetase (JAR1) gene suggested that JA was converted to its most bioactive compound, (-)-jasmonoyl-L-isoleucine (JA-Ile) under DS. Upon JA-Ile perception, the F-box protein coronatine-insensitive protein 1 (COI1) combined with jasmonate ZIM domain-containing protein (JAZ) repressors for ubiquitination and degradation by the ubiquitin-conjugating enzyme E2. This might lead to activation of TFs such as bHLH, MYB, and WRKY (S5C Fig). The v-myc myelocytomatosis viral oncogene homolog 2 (MYC2) is an important regulator of various JA responses and mediates crosstalk with other pathways [40]. However, in our study, only three *MYC* genes including one *MYC2* were up-regulated in tea plant under DS. More *MYB* and *WRKY* genes were activated, indicating that JA responses were under complex regulation by various TFs in tea plant [41].

In plants, ET is another signaling molecule that plays roles in drought tolerance [42]. Upon DS, the 1-aminocyclopropane-1-carboxylic acid (ACC) gene was up-regulated, and the *ACO* was also slightly up-regulated, indicating that DS induced the accumulation of ET. Genes encoding core components of ET signaling were also up-regulated during DS, activating diverse ET responses. During RC, genes related to ET synthesis and signaling were down-regulated. It had reported that ET production contributed to drought response in rice (*Oryza sativa*) [43]. These results suggested that ET may also be an important drought-responsive hormone in tea plant.

Intracellular IAA is synthesized from indole via tryptophan (Trp) or other intermediates [44]. In higher plants, there are three pathways for the biosynthesis of IAA from Trp: IAN, IAOx and IPA pathways [44,45]. Our results suggested that the IAOx and IPA pathways were activated for IAA biosynthesis under DS, while the IAN pathway was activated during RC (S5E Fig). Tea plant under DS might employ different pathways for IAA synthesis to avoid the reduction of IAA, thus maintaining active physiological processes to cope with DS. Mahouachi *et al*. (2007) reported that no significant changes in IAA levels were observed in papaya (*Carica papaya*.) seedling leaves during water stress and rewatering [46]. However, genes related to IAA signal transduction were down-regulated under DS, while up-regulated under RC, indicating that IAA signaling in tea plant was influenced by DS.

The natural CKs commonly found in higher plants are mainly *trans*-zeatin, *cis*-zeatin (low or no activity), and isopentenyladenine [47]. Unexpectedly, the genes encoding enzymes involved in CKs biosynthesis were up-regulated to some extent under DS, but down-regulated during RC (S5F Fig). The metabolism of purines, especially adenine, yields cytokinin precursors including dimethylallyl pyrophosphate (DMAPP), adenosine triphosphate (ATP), adenosine diphosphate (ADP), and adenosine monophosphate (AMP) [48,49]. The results of the KEGG pathway enrichment analysis showed that purine metabolism was significantly enriched under DS, but reduced during RC. It has been reported that elevated cytokinin levels can partially alleviate the negative effects of stress on photosynthetic activity and suppress stress-accelerated senescence of older and mature leaves [47]. These results, combined with our findings, have at least partly unraveled the mechanism of cytokinin metabolism in tea plant during DS and RC.

Protein kinases and protein phosphatases often act in tandem to phosphorylate and dephosphorylate their targets, thereby maintaining drought-signaling homeostasis in plants [50–52]. Several lines of evidence have suggested that PP2CA dephosphorylates the plant-specific ABA-activated SnRK2, inhibiting ABA signal transduction [51,52]. Furthermore, AtRbohD had been proved to be synergistically activated by Ca^2+^ and phosphorylation, and ROS signal transduction was modulated by phosphorylation [53]. Our study showed that different families of 26 protein kinase families had positive regulatory roles in responding to DS and RC, while the corresponding protein phosphatases played adverse regulatory roles, leading to maintain homeostasis of drought stress and water signal transduction in tea plant. After rehydration, the eight classes of up-regulated protein kinases might play essential roles in tea plant growth and development during RC.

### Transcription factors and other regulatory factors responding to dehydration and rehydration

Recent studies have demonstrated that several TFs have central roles in drought transcriptional regulation [54,55]. However, not all TFs were involved in the regulation of the responses to DS. Our data showed that the number of up-regulated TFs was less than down-regulated TFs during and after DS, and 12 TF families (AP2/EREBP, bHLH, bZIP, HD-ZIP, HSF, MYB, NAC, WRKY, zinc-finger protein TFs, SCL, ARR, and SPL) might play crucial roles in tea plant responding to DS. Our results were consistent with previous studies [54,56], which indicated that AP2/EREBP, bHLH, bZIP, HD-ZIP, HSF, MYB, NAC, WRKY, and zinc-finger protein TFs had vital roles in the plant response to DS. In poplar (*Populus euphratica*), SCL7 was induced by drought, and the drought tolerance of transgenic *Arabidopsis* overexpressing SCL7 was enhanced [57]. SCL14 was shown to play important roles in regulating plant growth and development as well as in the response to abiotic stresses such as DS [58]. It was reported that inducible expression of *ARR22* promoted tolerance to drought in *Arabidopsis* [59]. However, SPLs have been shown to be mainly involved in plant growth and development and in light signaling [60]. Induction of SPLs may help to reduce the negative effects of DS on the growth and morphology of tea plant. After rewatering, most members of the AP2/EREBP, B3, bHLH, GRF, HD-ZIP, MYB, SCL, SPL, and TCP gene families were up-regulated, indicating that these TFs have crucial roles in the RC of tea plant after DS. These TFs have been shown to have multiple functions in growth and development, such as leaf development, flower symmetry, and shoot branching [61,62].

Our results showed that other regulatory factors such as phospholipase D, ubiquitin, and nitric oxide were also involved in drought resistance in tea plant (S9 Fig). These regulatory factors have been shown to activate the expressions of genes encoding protective proteins such as late embryogenesis-abundant proteins, aquaporins, dehydrin, thioredoxins, heat shock proteins, and ion channels [5]. Together, these gene products might contribute to the adaptation or resistance of tea plant to DS.

### Osmotic adjustment in response to drought stress and recovery

Sugars, sugar alcohols, and proline are the primary osmoprotective compounds that contribute to osmotic adjustment and resistance/adaptation to DS in plants [63,64]. In plants, carbon assimilation products are produced in the leaves via photosynthesis. Rubisco, a key enzyme in the Calvin cycle, assimilates atmospheric CO_2_ into the biosphere [65]. However, the underlying metabolic mechanism of these osmolytes remains elusive in tea plant during and after DS. In the present study, the Rubisco gene was down-regulated under DS and then up-regulated after rewatering, indicating that carbon fixation was inhibited under DS. Under DS and RC, significant changes in expression of the key enzyme genes invovled in non-soluble sugar (starch) and soluble sugars (mannitol, trehalose and sucrose) were observed. These results were in accordance with our previous studies [8], which indicated that the soluble sugars in tea plant increased significantly as DS progressed and then rapidly decreased following rehydration. These results suggested that under DS, photo-assimilated carbon was preferentially used to synthesize osmolytes, and starch was mainly degradated into glucose. These results also suggested that during RC, the soluble sugars content decreased, but the flow of carbon into starch increased.

Hexokinase (HXK) is a regulatory enzyme in the glycolytic pathway [66]. The increased transcript level of *HXK* under DS suggested that tea plant maintained the ATP supply by sustaining glycolytic metabolism under DS. After rehydration, a *HXK* expression was slightly down-regulated, compared with its transcript level under DS (S8A Fig).

Proline is synthesized from glutamate or ornithine [64]. Our data suggested that proline was mainly biosynthesized from glutamate in tea plant under drought and rewatering.

### ABA regulation of stomatal movement in tea plant during drought stress and recovery

Stomatal pores control gas exchange and transpirational water loss, and have essential roles in resistance to abiotic stresses such as DS [67,68]. Stomatal closure is mediated by the release of potassium and various anions from guard cells [68]. Under DS, ABA-activated SnRK2 phosphorylated RbohD, resulting in the production of ROS in tea plant (S10 Fig). Superoxide dismutase (SOD), the first line of defense against ROS, dismutates superoxide to H_2_O_2_. The activity of SOD increased under DS, and the resulting increase in H_2_O_2_ content activated Ca^2+^ channels, leading to increased Ca^2+^ influx. Then, Ca^2+^-activated calcium-dependent protein kinase (CDPK), CBL-interacting serine/threonine-protein kinase (CIPK), and mitogen-activated protein kinase (MAPK) regulated stomatal closure via a series of cascade responses, such as repressing the expressions of some genes [*KAT1* (K^+^ transporter of *Arabidopsis thaliana* 1), *PMCA5/8* (calcium-transporting ATPase 5/8, plasma membrane-type), and *PM H-ATPase* (plasma membrane H^+^-ATPase)] and up-regulating expressions of other genes [*GORK* (gated outwardly-rectifying K^+^ channel), *ALMT12* (aluminum-activated malate transporter 12, an R-type anion channel in guard cells), and *V H-ATPase* (vacuolar H^+^-ATPase)] (S10 Fig). SnRK2 was shown to directly regulate the expressions of *KAT1*, *GORK*, *ALMT12*, and *PM H-ATPase* [7,69]. Under DS, Ca^2+^ stored in the endoplasmic reticulum and vacuole was probably transferred to the cytosol to enhance Ca^2+^ signaling by increasing the activities of ECA1/2 (Ca^2+^-ATPase 1/2, endoplasmic reticulum-type) and the V H-ATPase, and decreasing the activity of CAX1 (Ca^2+^/H^+^ antiporter 1). During RC, the decrease in ABA content might lead to decreased Ca^2+^ levels in the cytosol, which resulted in increased influx of potassium and various anions into guard cells. This might stimulate stomatal opening, thereby contributing to tea plant returning to normal growth (S10 Fig).

### Conclusions

Drought is a major constraint for the growth, yield, and quality of tea plant. Given that little genomic data are available for this species, and based on our previous morphological and physiological analyses [9], five reliable tea samples (CK, DS, and RC) were used for RNA-Seq to reveal novel responses to DS and RC. A series of candidate genes involved in the DS response were identified. Signaling and metabolic pathways and regulation of TFs involved in dehydration and rehydration were explored at the global transcriptional level. Our data suggested that calcium signaling and stomatal movement are important in the DS and RC responses in tea plant. Our results provide valuable information on the molecular responses of tea plant to DS and RC.

## Supporting Information

S1 Fig Overview of unigenes assembly by Trinity. (A) Length distribution of contigs obtained from *de novo* assembly of high-quality clean reads. (B) Length distribution of unigenes produced by joining contigs.
(TIF)

S2 Fig Transcriptome coding sequences (CDSs) predicted by BLASTx and ESTScan. (A) Length distribution of CDSs using BLASTx. (B) Length distribution of proteins using BLASTx. (C) Length distribution of CDSs using ESTscan. (D) Length distribution of proteins using ESTscan.
(TIF)

S3 Fig Enrichment of KEGG pathways in tea plant under dehydration and rehydration. Categories of enriched pathways shown on x-axis; bottom y-axis shows number of genes in each pathway; top y-axis represents −log_10_^(corrected P-valure)^ value of pathway enrichment (where larger value of −log_10_^(corrected P-value)^ indicates more significant pathway enrichment). When the corrected *p*-value was < 0.05, −log_10_^0.05^ >1.301, the KEGG pathway was significantly enriched.
(TIF)

S4 Fig Validation of RNA-Seq results using qRT-PCR. Twenty unique genes with markedly altered expression patterns in response to dehydration and rehydration were selected from among signal component, transcription factor, biochemical pathway, and functional genes. qRT-PCR data were normalized against ‘housekeeping’ gene *GAPDH*.
(TIF)

S5 Fig The putative pathways of phytohormone metabolism and signaling during and after drought stress. (A) Abscisic acid (ABA) metabolism and signaling in tea plant. ABF, ABRE-binding factor; ABA-GE, ABA glucose ester; ABA2, xanthoxin dehydrogenase; AOG, abscisate β-glucosyltransferase; BG1, β-D-glucopyranosyl abscisate β-glucosidase; CPY707A, (+)-abscisic acid 8'-hydroxylase; NCED, 9-*cis*-epoxycarotenoid dioxygenase; PA, phaseic acid; PP2C, protein phosphatase 2C; PYL, abscisic acid receptor PYL family; RBOHD, Respiratory burst oxidase homolog D; SnRK2, sucrose non-fermenting 1-related protein kinase 2; ZEP, zeaxanthin epoxidase. (B) Salicylic acid (SA) metabolism and signaling. ICS, isochorismate synthase; NPR1, nonexpressor of pathogenesis-related genes 1; PAL, phenylalanine ammonia-lyase; PR, pathogenesis-related protein; SNC1, suppressor of npr1-1, constitutive 1; TGA, bZIP transcription factor TGA. (C) Jasmonic acid (JA) metabolism and signaling. ACOX, acyl-CoA oxidase; AOC, allene oxide cyclase; AOS, allene oxide synthase; bHLH, basic helix-loop-helix protein; COI1, coronatine-insensitive protein 1; E2, ubiquitin-conjugating enzyme; 12,13-EOT, 13S-12,13-epoxyoctadeca-9,11,15-trienoic acid; fadA, acetyl-CoA acyltransferase; HPL, hydroperoxide lyase; 13-HPOT, 13-hydroperoxy-9,11,15-octadecatrienoic acid; JA-Ile, (-)-Jasmonoyl-L-isoleucine; JAR1, jasmonic acid-amino synthetase; JAZ, jasmonate ZIM domain-containing protein; α-LeA, α-Linolenic acid; LOX, lipoxygenase; OPCL1, OPC-8:0 CoA ligase 1; OPDA, (15Z)-12-oxophyto-10,15-dienoate; OPC-8:0, 8-[(1R,2R)-3-Oxo-2-{(Z)-pent-2-enyl}cyclopentyl] octanoate; OPR,12-oxophytodienoic acid reductase; PC, phosphatidylcholine; PLA1, phospholipase A1; TA, traumatic acid. (D) Ethylene metabolism and signaling. ACC, 1-Aminocyclopropane-1-carboxylic acid; ACO, aminocyclopropanecarboxylate oxidase; ACS, 1-aminocyclopropane-1-carboxylate synthase; CTR1, serine/threonine-protein kinase CTR1; EBF1/2, EIN3-binding F-box protein; EIN2/3, ethylene-insensitive protein 2/3; ER, endoplasmic reticulum; ETR, ethylene receptor; MTA, 5-methylthioadenosine; SAM, S-adenosylmethionine. (E) Indole-3-acetic acid (IAA) metabolism and signaling. AAO1_2, indole-3-acetaldehyde oxidase; ALDH, aldehyde dehydrogenase (NAD+); ARF, auxin response factor; AUX1, auxin influx carrier; AUX/IAA, auxin-responsive protein IAA; GH3, the auxin-responsive Gretchen Hagen3 gene family; IAA1d, indole-3-acetaldehyde; IAN, 3-indoleacetonitrile; IAOx, 3-indoleacetaldoxime; IPA, indole-3-pyruvic acid; NIT, nitrilase; SAUR, small auxin upregulated RNA; TAM, tryptamine; TIR1, transport inhibitor response 1; Ub, ubiquitylation. (F) Cytokinin (CK) metabolism and signaling. AHKs, *Arabidopsis* histidine kinases; AHPs, *Arabidopsis* histidine phosphotransfer proteins; ARR-A, type-A *Arabidopsis* response regulator; ARR-B, type-B *Arabidopsis* response regulator; CKX, CK dehydrogenase; CYP735A, cytochrome P450 monooxygenase 735A; DMAPP, dimethylallyl diphosphate; iP, isopentenyladenine; iPDP, isopentenyl-ADP; iPMP, isopentenyl-AMP; iPR, isopentenyladenine riboside; iPTP, isopentenyl-ATP; IPT, isopentenyltransferase; *t*ZRDP, *trans*-zeatin riboside diphosphate; *t*ZRMP, *trans*-zeatin riboside monophosphate; *t*ZRTP, *trans*-zeatin riboside triphosphate; *t*ZR, *trans*-zeatin riboside; *t*ZT, *trans*-zeatin.
(TIF)

S6 Fig Different responses of protein kinases and protein phosphatases during and after drought stress. Within each bar, number of up- and down-regulated genes is shown in red and blue, respectively. Details are not shown for protein kinase and protein phosphatase families with fewer than six members.
(TIF)

S7 Fig Transcription factors (TFs) responsive to drought stress and rewatering. Within each bar, number of up- and down-regulated genes is shown in red and blue, respectively. Details are not shown for TF families with fewer than six members.
(TIF)

S8 Fig Predicted metabolism of non-structural carbohydrate and proline in tea plant under dehydration and rehydration. (A) Non-structural carbohydrate metabolism. AAMY, α-amylase; ADPG, ADP-glucose; AGPase, ADP-glucose pyrophosphorylase; BAMY, β-amylase; Fru, fructose; F6P, fructose-6-phosphate; Glc, glucose; G1P, glucose-1-phosphate; G6P, glucose-6-phosphate; HXK, hexokinase; M1PP, mannose-1 phosphate phosphatase; M6PR, mannose-6-phosphate reductase; Rubisco, ribulose-1,5-bisphosphate carboxylase; SP, starch phosphorylase; SPP, sucrose-6-phosphate phosphohydrolase; SPS, sucrose-phosphate synthase; SS, starch synthase; SuS, sucrose synthase; 3-PGA, 3-phosphoglycerate; TPP, trehalose phosphatases; TPS, trehalose phosphate synthase; Tre, trehalase; Tre6P, trehalose-6-phosphate; UDPGase, UDP-glucose pyrophosphorylase; UDPG, UDP-glucose. (B) Proline metabolism. G5SA, L-Glutamate-5-semialdehyde; Glup, L-Glutamyl-5-phosphate; GRR, γ-glutamyl phosphate reductase; OAT, ornithine amino transferase; P5CS, pyrroline-5-carboxylate synthetase; P5C, 1-Pyrroline-5-carboxylate; P5CR, pyrroline-5-carboxylate reductase; ProDH, proline dehydrogenase.
(TIF)

S9 Fig Other potential regulatory factors responding to dehydration and rehydration. (TIF)

S10 Fig Potential regulation of abscisic acid-induced stomatal movement during drought stress and recovery. ALMT12, aluminum-activated malate transporter 12; CaM, calmodulin; CAX1, Ca^2+^/H^+^ antiporter 1; CBL1, calcineurin B-like protein 1; ECA1/2, Ca^2+^-ATPase 1/2, endoplasmic reticulum-type; ER, endoplasmic reticulum; GORK, gated outwardly-rectifying K^+^ channel; KAT1, K^+^ transporter of *Arabidopsis thaliana* 1; PMCA5/8, calcium-transporting ATPase 5/8, plasma membrane-type; PM H-ATPase, plasma membrane H^+^-ATPase; SOD, superoxide dismutase; TPC1A/B, two pore calcium channel protein 1 A/B; V H-ATPase, vacuolar H^+^-ATPase.
(TIF)

S1 Table Primers used for qRT-PCR analyses. Primers listed were used to amplify 20 genes that were randomly selected for qRT-PCR anazlyses to validate DEG reliability and ‘housekeeping’ gene *GAPDH* used to quantify gene expression.
(DOC)
