# Supplementary figures and images for: Transcriptomic Analysis of Tea Plant Responding to Drought Stress and Recovery
Source: PLoS One. 2016 Jan 20;11(1):e0147306. doi: 10.1371/journal.pone.0147306 (PMC4720391; doi:10.1371/journal.pone.0147306)

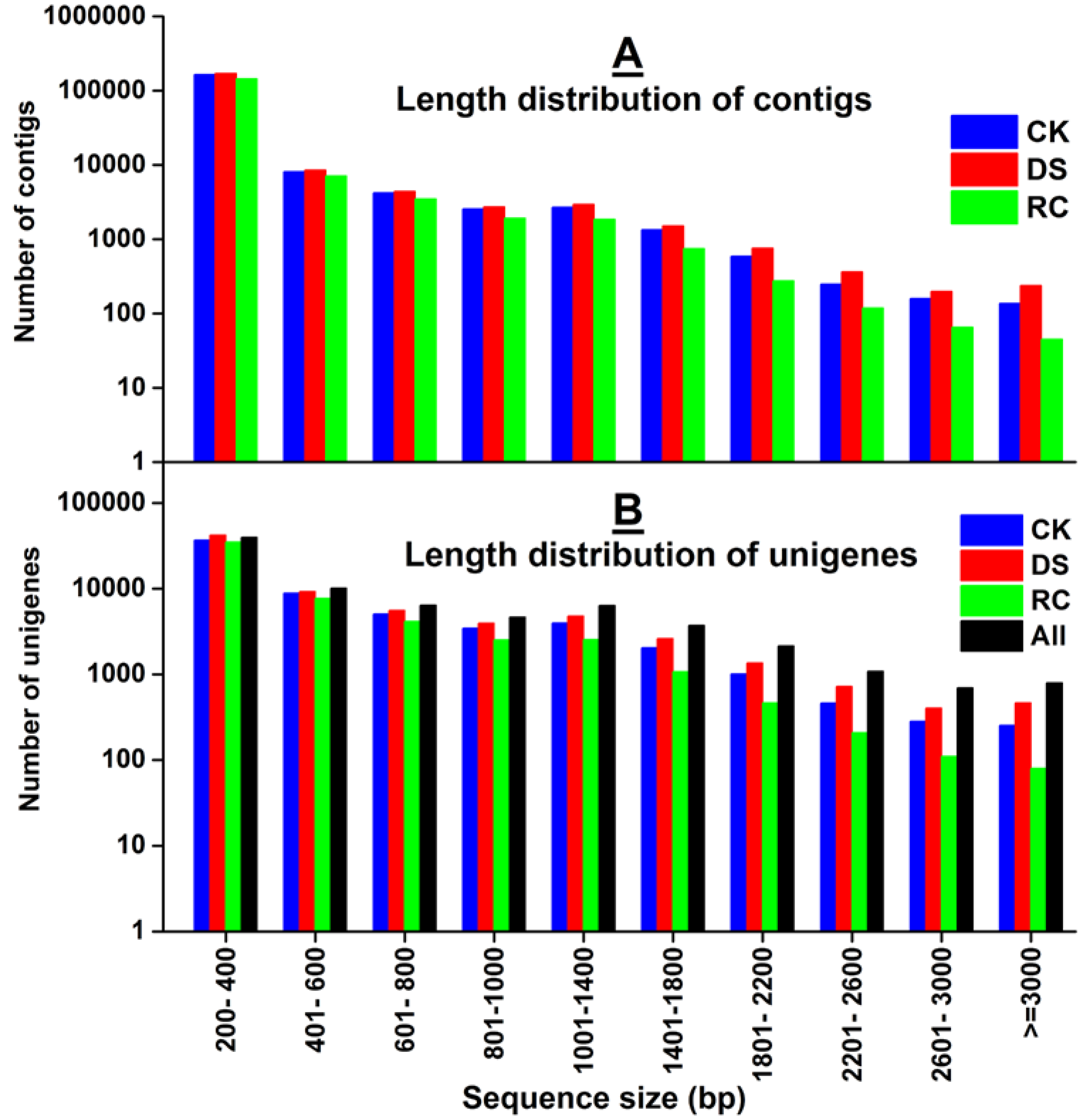

Supplement: S1 Fig — (A) Length distribution of contigs obtained from de novo assembly of high-quality clean reads. (B) Length distribution of unigenes produced by joining contigs. (TIF) [file pone.0147306.s001.tif]

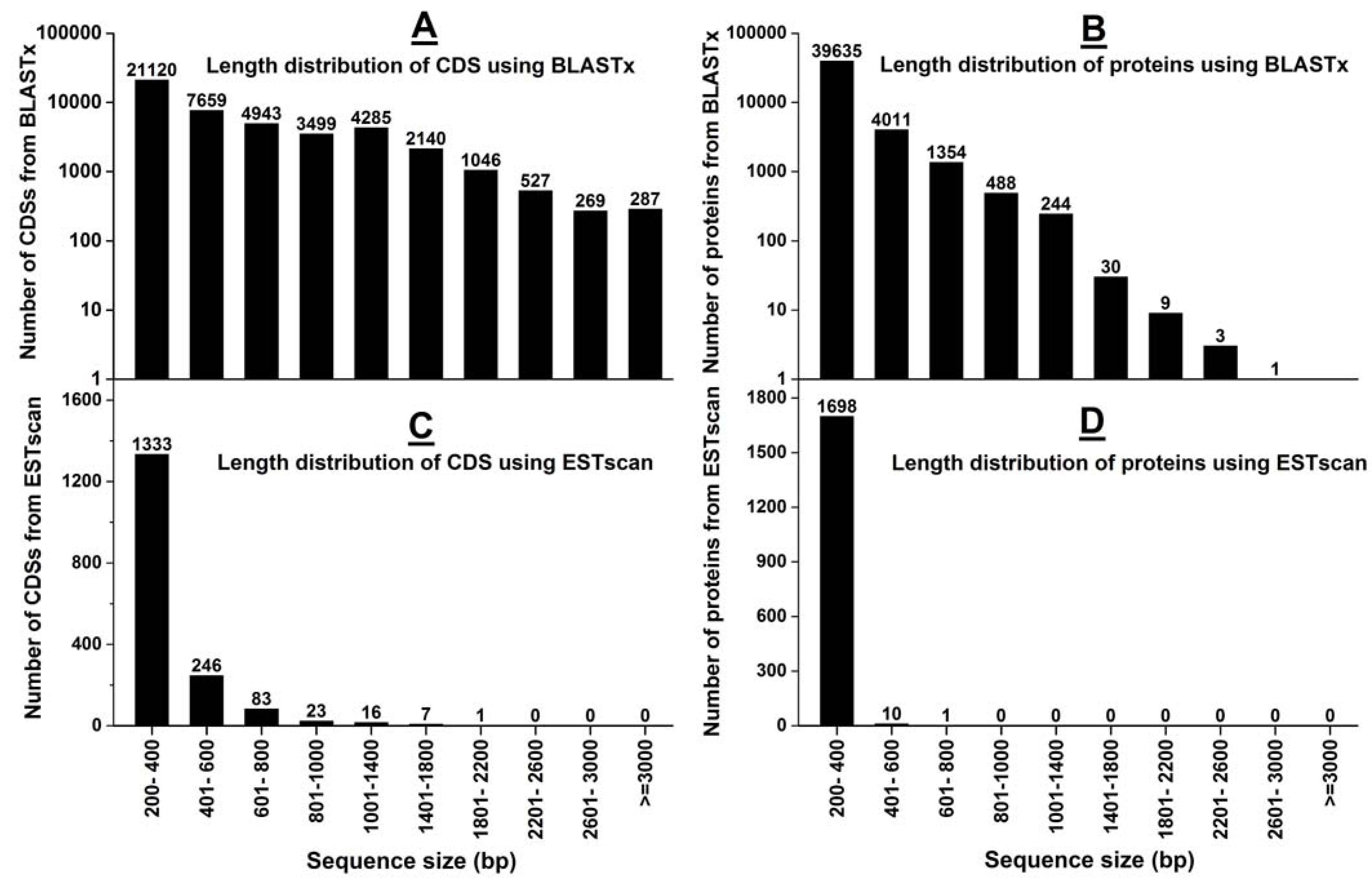

Supplement: S2 Fig — (A) Length distribution of CDSs using BLASTx. (B) Length distribution of proteins using BLASTx. (C) Length distribution of CDSs using ESTscan. (D) Length distribution of proteins using ESTscan. (TIF) [file pone.0147306.s002.tif]

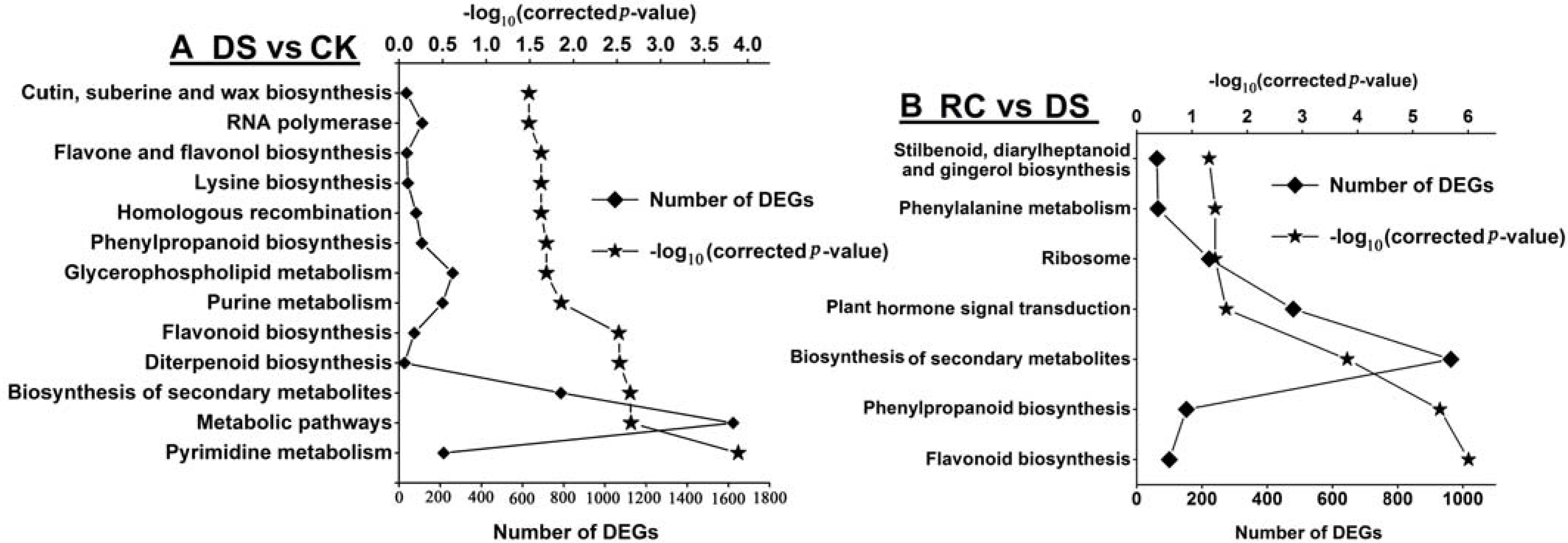

Supplement: S3 Fig — Categories of enriched pathways shown on x-axis; bottom y-axis shows number of genes in each pathway; top y-axis represents −log10(corrected P-valure) value of pathway enrichment (where larger value of −log10(corrected P-value) indicates more significant pathway enrichment). When the corrected p-value was < 0.05, −log100.05 >1.301, the KEGG pathway was significantly enriched. (TIF) [file pone.0147306.s003.tif]

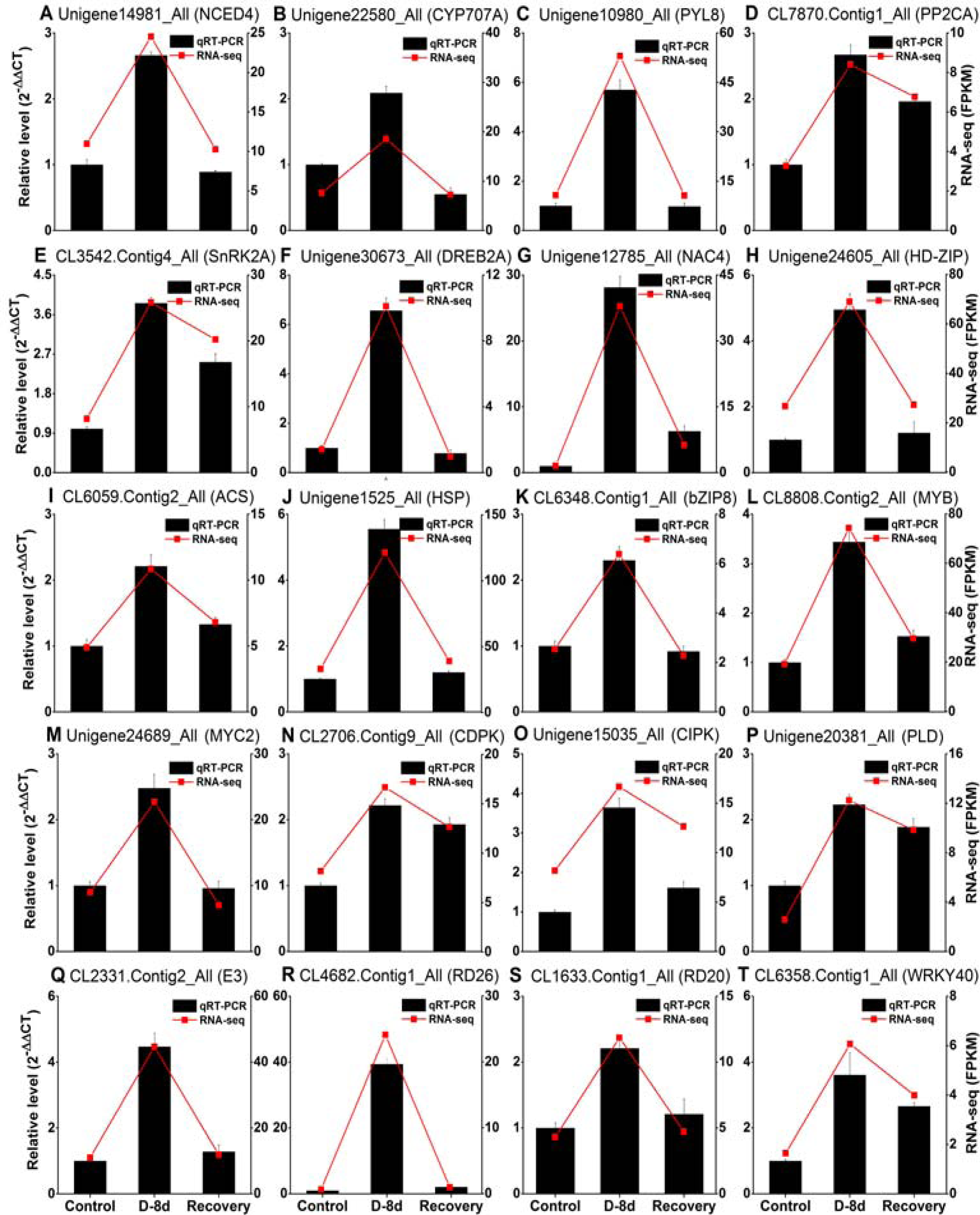

Supplement: S4 Fig — Twenty unique genes with markedly altered expression patterns in response to dehydration and rehydration were selected from among signal component, transcription factor, biochemical pathway, and functional genes. qRT-PCR data were normalized against ‘housekeeping’ gene GAPDH. (TIF) [file pone.0147306.s004.tif]

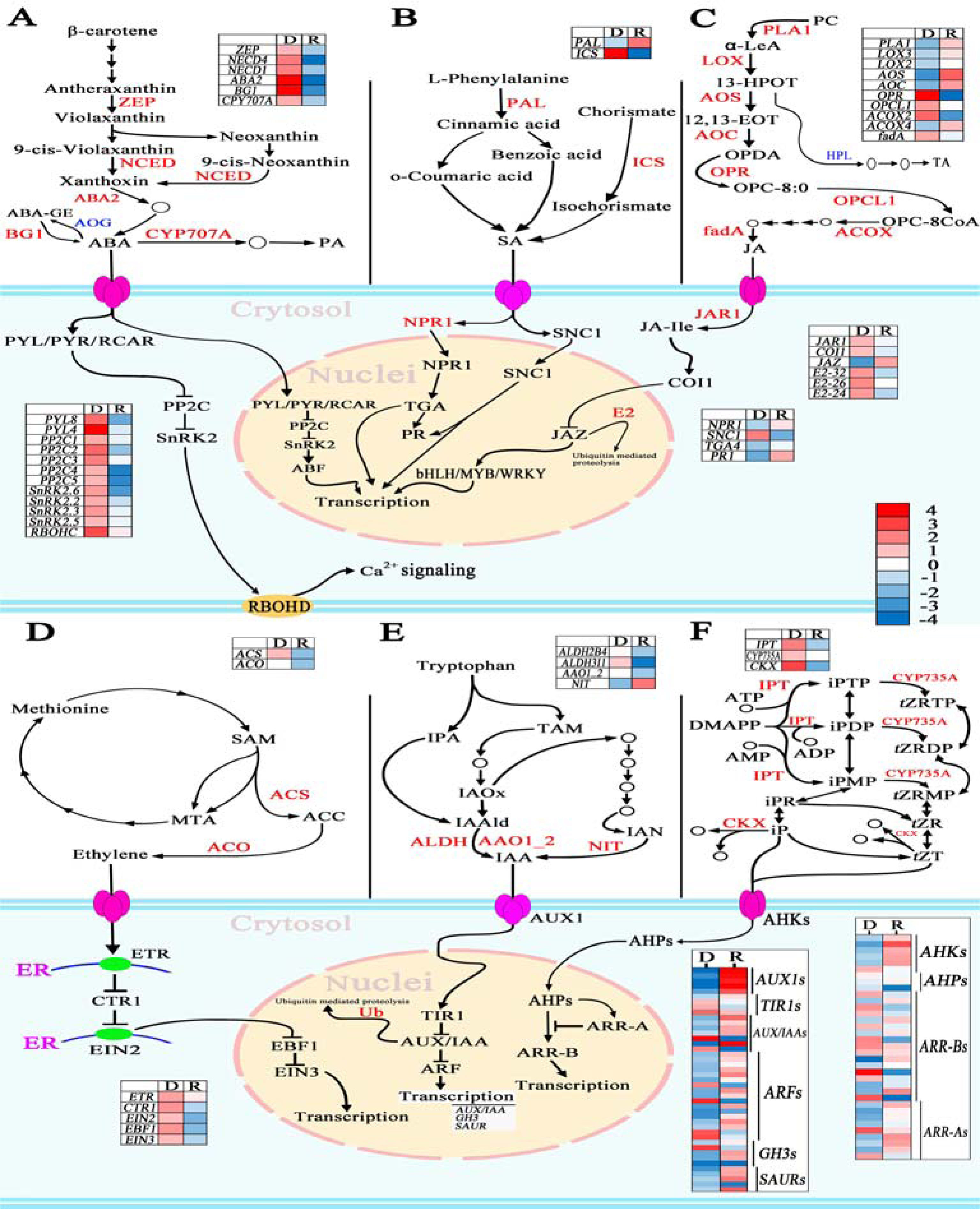

Supplement: S5 Fig — (A) Abscisic acid (ABA) metabolism and signaling in tea plant. ABF, ABRE-binding factor; ABA-GE, ABA glucose ester; ABA2, xanthoxin dehydrogenase; AOG, abscisate β-glucosyltransferase; BG1, β-D-glucopyranosyl abscisate β-glucosidase; CPY707A, (+)-abscisic acid 8'-hydroxylase; NCED, 9-cis-epoxycarotenoid dioxygenase; PA, phaseic acid; PP2C, protein phosphatase 2C; PYL, abscisic acid receptor PYL family; RBOHD, Respiratory burst oxidase homolog D; SnRK2, sucrose non-fermenting 1-related protein kinase 2; ZEP, zeaxanthin epoxidase. (B) Salicylic acid (SA) metabolism and signaling. ICS, isochorismate synthase; NPR1, nonexpressor of pathogenesis-related genes 1; PAL, phenylalanine ammonia-lyase; PR, pathogenesis-related protein; SNC1, suppressor of npr1-1, constitutive 1; TGA, bZIP transcription factor TGA. (C) Jasmonic acid (JA) metabolism and signaling. ACOX, acyl-CoA oxidase; AOC, allene oxide cyclase; AOS, allene oxide synthase; bHLH, basic helix-loop-helix protein; COI1, coronatine-insensitive protein 1; E2, ubiquitin-conjugating enzyme; 12,13-EOT, 13S-12,13-epoxyoctadeca-9,11,15-trienoic acid; fadA, acetyl-CoA acyltransferase; HPL, hydroperoxide lyase; 13-HPOT, 13-hydroperoxy-9,11,15-octadecatrienoic acid; JA-Ile, (-)-Jasmonoyl-L-isoleucine; JAR1, jasmonic acid-amino synthetase; JAZ, jasmonate ZIM domain-containing protein; α-LeA, α-Linolenic acid; LOX, lipoxygenase; OPCL1, OPC-8:0 CoA ligase 1; OPDA, (15Z)-12-oxophyto-10,15-dienoate; OPC-8:0, 8-[(1R,2R)-3-Oxo-2-{(Z)-pent-2-enyl}cyclopentyl] octanoate; OPR,12-oxophytodienoic acid reductase; PC, phosphatidylcholine; PLA1, phospholipase A1; TA, traumatic acid. (D) Ethylene metabolism and signaling. ACC, 1-Aminocyclopropane-1-carboxylic acid; ACO, aminocyclopropanecarboxylate oxidase; ACS, 1-aminocyclopropane-1-carboxylate synthase; CTR1, serine/threonine-protein kinase CTR1; EBF1/2, EIN3-binding F-box protein; EIN2/3, ethylene-insensitive protein 2/3; ER, endoplasmic reticulum; ETR, ethylene receptor; MTA, 5-methylth [file pone.0147306.s005.tif]

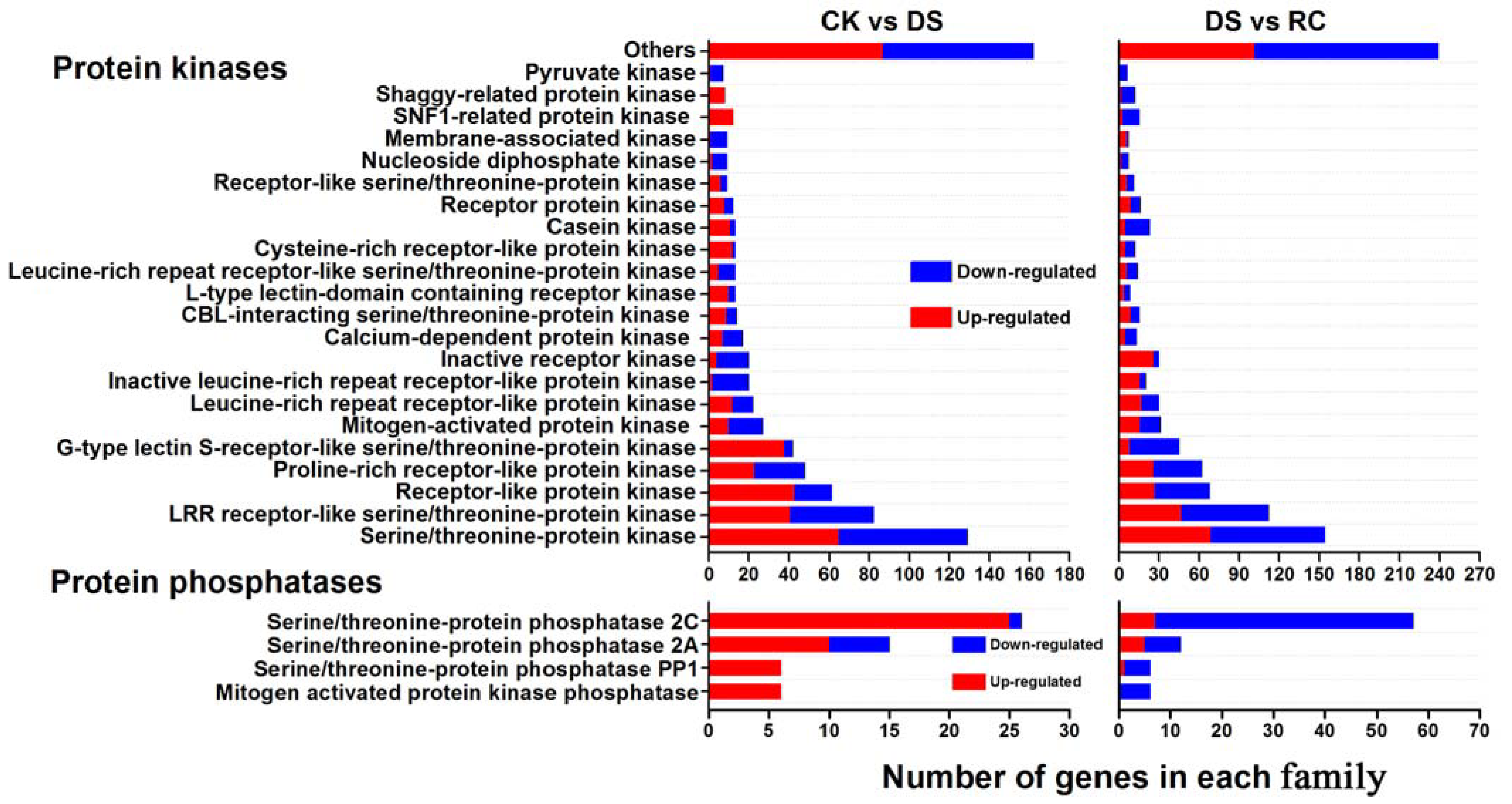

Supplement: S6 Fig — Within each bar, number of up- and down-regulated genes is shown in red and blue, respectively. Details are not shown for protein kinase and protein phosphatase families with fewer than six members. (TIF) [file pone.0147306.s006.tif]

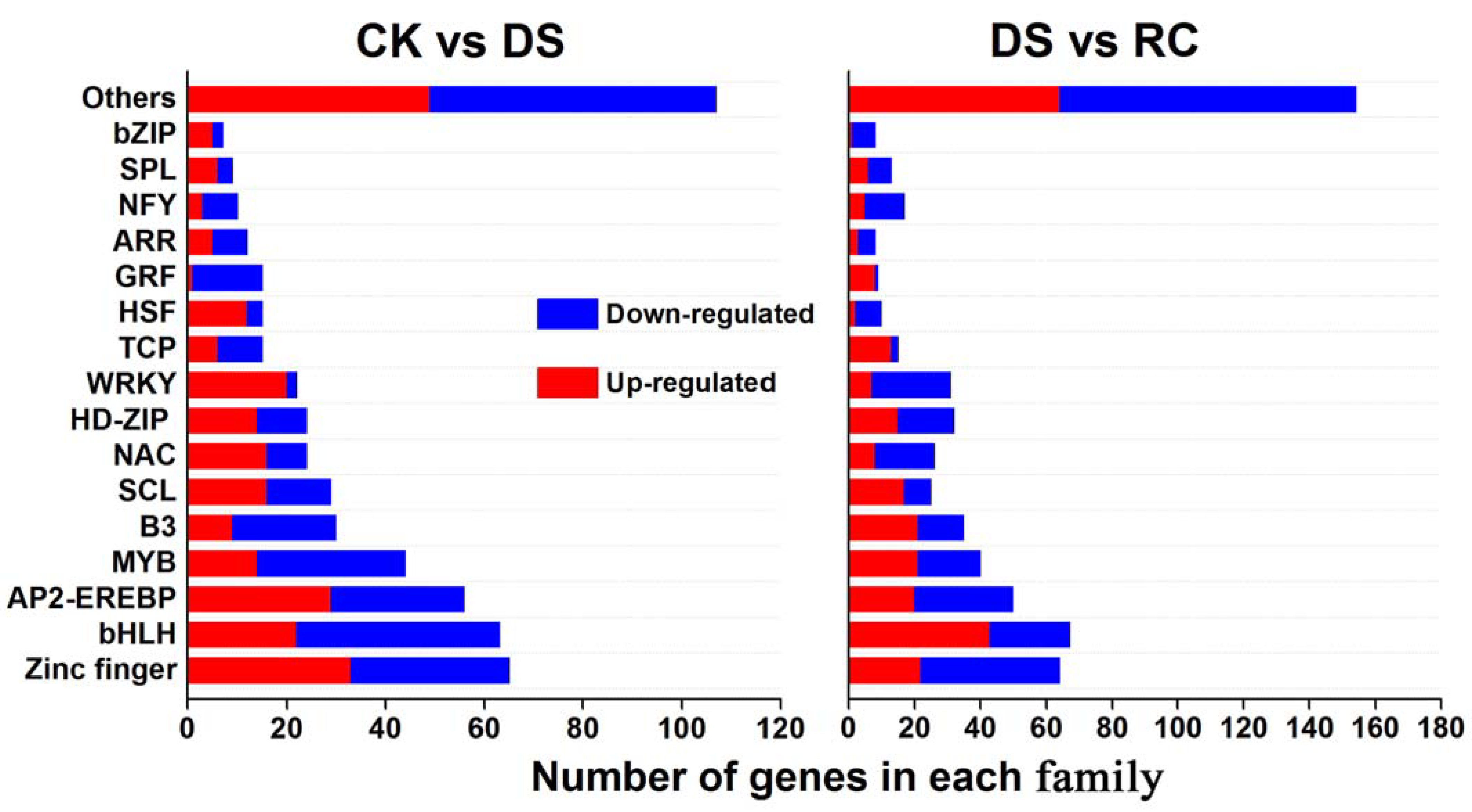

Supplement: S7 Fig — Within each bar, number of up- and down-regulated genes is shown in red and blue, respectively. Details are not shown for TF families with fewer than six members. (TIF) [file pone.0147306.s007.tif]

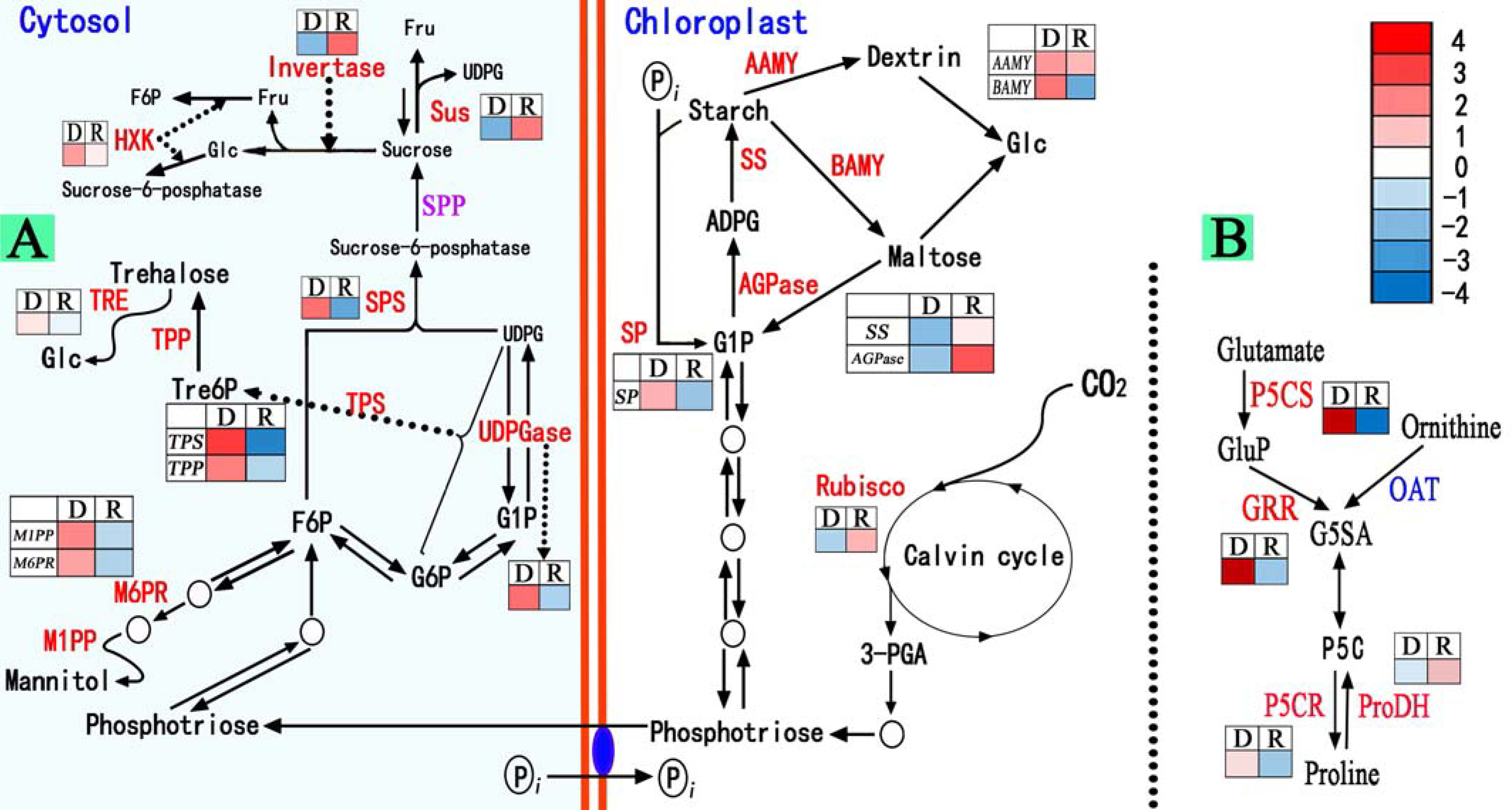

Supplement: S8 Fig — (A) Non-structural carbohydrate metabolism. AAMY, α-amylase; ADPG, ADP-glucose; AGPase, ADP-glucose pyrophosphorylase; BAMY, β-amylase; Fru, fructose; F6P, fructose-6-phosphate; Glc, glucose; G1P, glucose-1-phosphate; G6P, glucose-6-phosphate; HXK, hexokinase; M1PP, mannose-1 phosphate phosphatase; M6PR, mannose-6-phosphate reductase; Rubisco, ribulose-1,5-bisphosphate carboxylase; SP, starch phosphorylase; SPP, sucrose-6-phosphate phosphohydrolase; SPS, sucrose-phosphate synthase; SS, starch synthase; SuS, sucrose synthase; 3-PGA, 3-phosphoglycerate; TPP, trehalose phosphatases; TPS, trehalose phosphate synthase; Tre, trehalase; Tre6P, trehalose-6-phosphate; UDPGase, UDP-glucose pyrophosphorylase; UDPG, UDP-glucose. (B) Proline metabolism. G5SA, L-Glutamate-5-semialdehyde; Glup, L-Glutamyl-5-phosphate; GRR, γ-glutamyl phosphate reductase; OAT, ornithine amino transferase; P5CS, pyrroline-5-carboxylate synthetase; P5C, 1-Pyrroline-5-carboxylate; P5CR, pyrroline-5-carboxylate reductase; ProDH, proline dehydrogenase. (TIF) [file pone.0147306.s008.tif]

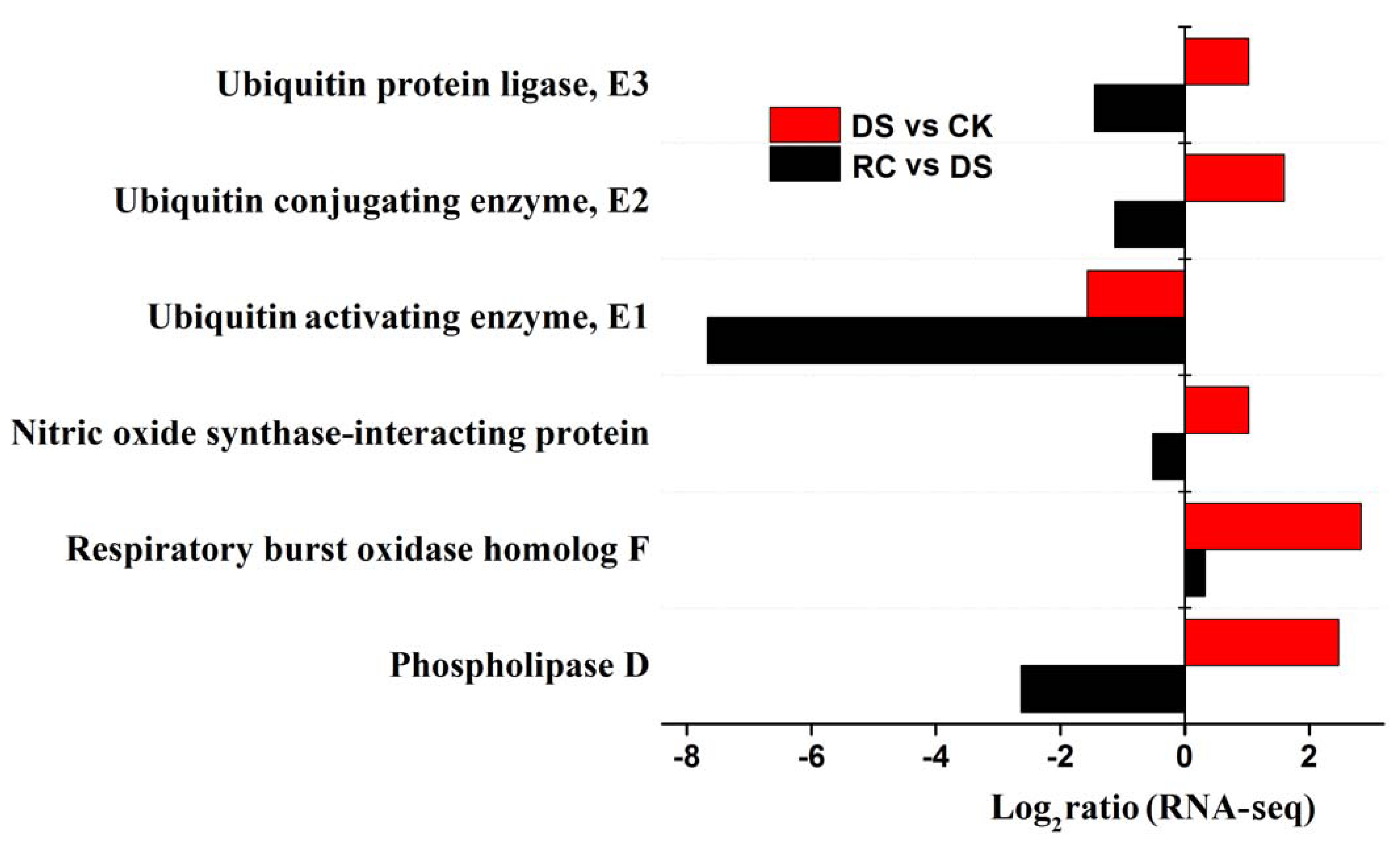

Supplement: S9 Fig — (TIF) [file pone.0147306.s009.tif]

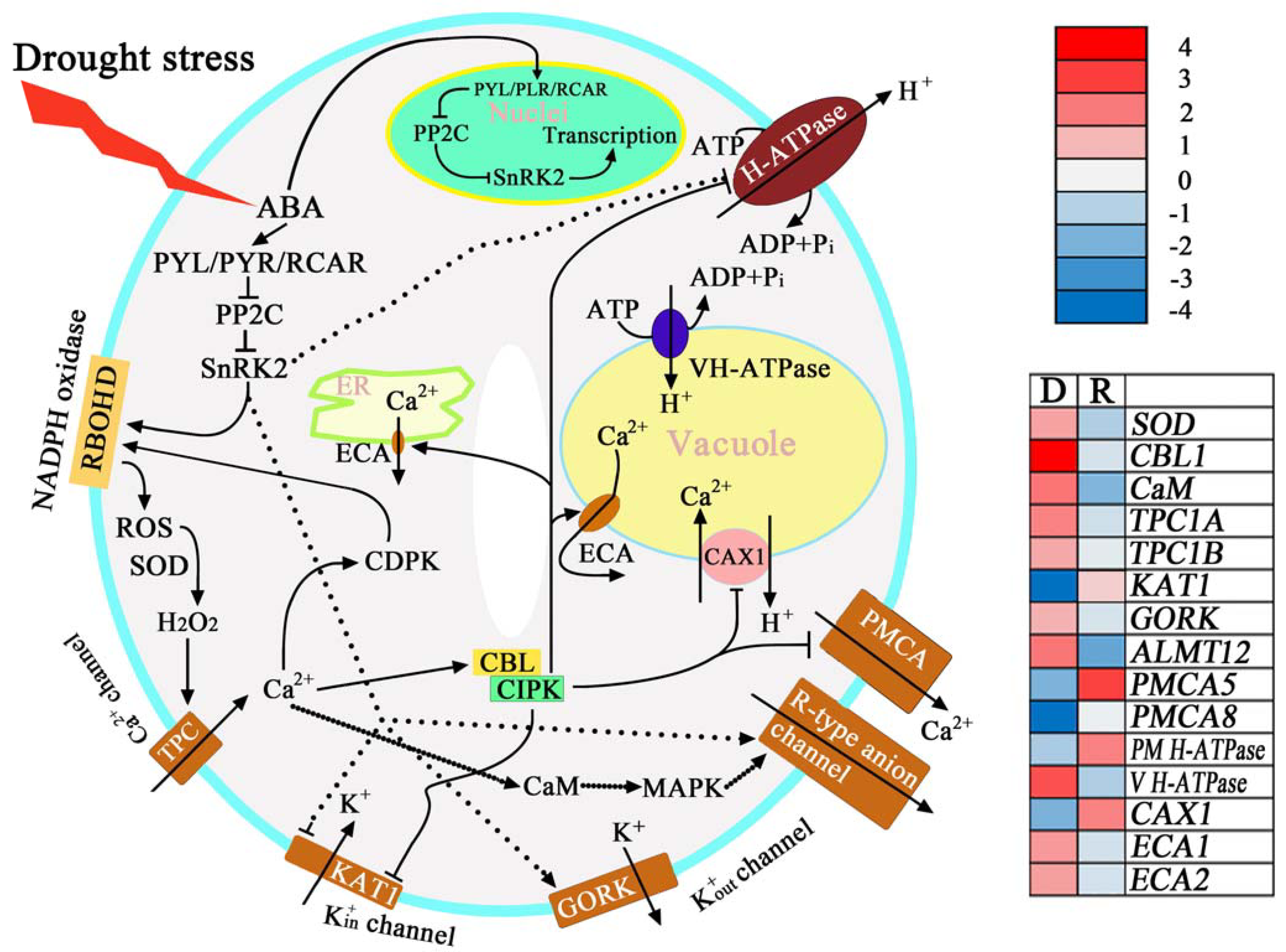

Supplement: S10 Fig — ALMT12, aluminum-activated malate transporter 12; CaM, calmodulin; CAX1, Ca2+/H+ antiporter 1; CBL1, calcineurin B-like protein 1; ECA1/2, Ca2+-ATPase 1/2, endoplasmic reticulum-type; ER, endoplasmic reticulum; GORK, gated outwardly-rectifying K+ channel; KAT1, K+ transporter of Arabidopsis thaliana 1; PMCA5/8, calcium-transporting ATPase 5/8, plasma membrane-type; PM H-ATPase, plasma membrane H+-ATPase; SOD, superoxide dismutase; TPC1A/B, two pore calcium channel protein 1 A/B; V H-ATPase, vacuolar H+-ATPase. (TIF) [file pone.0147306.s010.tif]
