# Supplementary material for: Transcriptomic Analysis of Tea Plant Responding to Drought Stress and Recovery
Source: PLoS One. 2016 Jan 20;11(1):e0147306. doi: 10.1371/journal.pone.0147306 (PMC4720391; doi:10.1371/journal.pone.0147306)
Supplement: S1 Table — Primers listed were used to amplify 20 genes that were randomly selected for qRT-PCR anazlyses to validate DEG reliability and ‘housekeeping’ gene GAPDH used to quantify gene expression. (DOC) [file pone.0147306.s011.doc]

**S1 Table. Primers used for qRT-PCR analyses**

| Gene ID | Primer sequence | Product length (bp) |
| --- | --- | --- |
| *GAPDH* | F: TTG GCA TCG TTG AGG GTC T  R: CAG TGG GAA CAC GGA AAG C | 206 |
| *Unigene14981 (NCED4)* | F: ATTTTGATGGCGTGGAGC  R: CGAACTCCCTCCGACCA | 149 |
| *Unigene22580 (CYP707A)* | F: GGAAATGAAATACACCGAGGCT  R: CGTAGACCGATGGGAACACG | 136 |
| *Unigene10980 (PYL8)* | F: CAGCACCGAAAGATTGGAAC  R: GACGGAAATGATTGAGGAGTAG | 152 |
| *CL7870.Contig1 (PP2CA)* | F: GTTTGTCACATTTGAAGTCGGAGAT  R: GAGTGCGGGAAAGCAGTTGAG | 156 |
| *CL3542.Contig4 (SnRK2A )* | F: AATATGCGACTTCGGCTACTC  R: ACATCTGCAATCTTCCCATCA | 125 |
| *Unigene30673 (DREB2A)* | F: ACACCGCCGTCTACTACCTCC  R: GCTCCGACCTCGGTTGCTTTT | 171 |
| *Unigene12785 (NAC4)* | F: CAATCTCAGGCTCAAACTCAA  R: CGAGTTCTCAAATCGGTGCTG | 107 |
| *Unigene24605 (HD-ZIP)* | F: TAGCACATCCGGTGACTCTGA  R: TGCTGAATGTTCTTGCCACTT | 123 |
| *CL6059.Contig2 (ACS)* | F: GATTGTACCCGTACCTTGCTG  R: CTGATTGAGAAGATTGCCCAC | 143 |
| *Unigene1525 (HSP)* | F: ATGCTGCTATTGAACTCCTTG  R: GCAAACGCTGTAACTTCTGTG | 151 |
| *CL6348.Contig1 ( bZIP8)* | F: TATTTCCTCCTGTTTCTTGCG  R: ATGCGGCAGAATGGTAATGTC | 156 |
| *CL8808.Contig2 (MYB)* | F: CAGGCAGAACAGATAATGAGG  R: ATCGTTTATTGGCTTGTGGGT | 152 |
| *Unigene24689 (MYC2)* | F: AAGGATGGTAAAGAGGGTGTT  R: TGAGAAGAAGGCAAGAAGAAC | 123 |
| *CL2706.Contig9 (CDPK)* | F: GGGACTGTTGAGCAGGTATTG  R: CGGCATAGGCGATTCAGGTTC | 145 |
| *Unigene15035 (CIPK)* | F: AAGCCAGGGAATGCTGTAAGA  R: CTCATCAAGAACAAATAAGGGT | 98 |
| *Unigene20381 (PLD)* | F: AGCCACGAAATGGTCAGAGTT  R: GATCATCTTCCCTGGAAACCC | 180 |
| *CL2331.Contig2 (E3)* | F: CAATGGGCAGGAAGAAATAAC  R: TAGCAATAGTCCGAGGGTGAG | 132 |
| *CL4682.Contig1 (RD26)* | F: ACAGAACCCAATCATCCAACT  R: ATTGGAAAGCCACTGGAACTG | 146 |
| *CL1633.Contig1 (RD20)* | F: TGCAAATGCCTTAACATCAGA  R: GGCTTCCATCATAAACGGCTC | 135 |
| *CL6358.Contig1 (WRKY40)* | F: AAATGGTGCTGAGGCTGAAAT  R: TGAAGGTACTCACAACCATCCC | 137 |
